# Hybrid Multimodal Surrogate Modeling and Uncertainty-Aware Co-Design for L-PBF Ti-6Al-4V with Nanomaterials-Informed Morphology Proxies

**DOI:** 10.3390/nano16080447

**Published:** 2026-04-08

**Authors:** Rifath Bin Hossain, Xuchao Pan, Geng Chang, Xin Su, Yu Tao, Xinyi Han

**Affiliations:** School of Mechanical Engineering, Nanjing University of Science and Technology, No. 200 Xiaolingwei Street, Nanjing 210094, China; rifath.bin.hossain@njust.edu.cn (R.B.H.); changgeng@njust.edu.cn (G.C.); taoyu@njust.edu.cn (Y.T.);

**Keywords:** laser powder bed fusion (L-PBF), Ti-6Al-4V, multimodal learning, morphology descriptors, uncertainty quantification, surrogate modeling, constrained co-design

## Abstract

Reliable property prediction and process selection in laser powder bed fusion are hindered by small, set-level datasets in which key morphology descriptors are intermittently missing, limiting both generalization and actionable co-design. A hybrid multimodal surrogate strategy is introduced that couples engineered process physics features with morphology proxies through a deployable two-stage embedding module and gradient-boosted tree regressors. Set-resolved inputs are assembled from L-PBF parameters, linear energy density and related energy-density variants, pore and prior-β grain summary statistics, and stress–strain-derived descriptors, followed by missingness-aware feature filtering, median imputation, and 5-fold GroupKFold evaluation grouped by set_id, with morphology embeddings learned on training folds and predicted when absent. Across six targets, the final deployable models achieve an RMSE/R^2^ of 11.07 MPa/0.895 (yield), 13.88 MPa/0.873 (UTS), 0.677%/0.861 (elongation), and 2.38 GPa/0.663 (modulus), while roughness and hardness remain challenging (RMSE 2.31 μm and 16.54 HV; R^2^ about 0.12 and 0.11). These surrogates enable constraint-aware candidate generation that identifies a concise set of manufacturing recipes balancing strength and surface objectives under uncertainty-aware screening. The resulting framework provides a practical blueprint for multimodal, small-data additive manufacturing studies and can be extended to richer microstructure measurements and prospective validation to accelerate functional and biomedical alloy development.

## 1. Introduction

The laser powder bed fusion of Ti-6Al-4V enables complex geometries and functional surfaces, but reliable qualification and rapid recipe development are still constrained by the difficulty of predicting performance from process settings alone [1,2,3]. The underlying scientific challenge is the coupled process of the structure to property pathway, where process variables and engineered energy-input descriptors act as proxies for thermal histories that shape defect populations and microstructural evolution, and these latent states mediate mechanical and surface responses [4,5,6,7,8,9]. A practical solution is to build recipe-level surrogate models that learn this mapping using a consistent experimental unit and statistically defensible evaluation while integrating morphology and proxy information when available and quantifying uncertainty for decision-making [10,11]. In this study, the learning problem is explicitly defined at the set level (set_id), and the dataset is treated as intrinsically multimodal by combining process and engineered physics features with pore, prior-β grain, and stress–strain-derived proxy blocks where available.

Research directly aligned with deployable, recipe-level, multimodal property prediction remains limited, and much of the literature addresses narrower subproblems. Early representative work focused on predicting porosity from process parameters using statistical models with Bayesian inference (for example, Tapia and Elwany, 2015) [12]. Subsequent work developed Gaussian process formulations for porosity prediction as a function of process parameters, including spatial Gaussian process regression variants [13]. A widely used modern pattern for efficient exploration is to couple Gaussian process regression with Bayesian optimization to guide sampling of the L-PBF process space, including demonstrations on Ti-6Al-4V that combine rapid characterization with model-guided discovery of processing domains [14]. In parallel, the community continues to debate the reliability of scalar energy-density compressions as general design variables, and recent reviews emphasize that volumetric energy density can correlate with outcomes in restricted settings while failing as a universal predictor when different parameter combinations yield different melt-pool regimes [15,16]. These directions are informative, but they do not by themselves resolve the deployment gap created by incomplete morphology availability, small recipe counts, and the need for uncertainty-aware co-design across multiple targets.

The present work addresses the deployment gap by structuring the entire pipeline around recipe-level learning, leakage control, modality heterogeneity, and decision-time constraints, rather than assuming retrospective access to all measurements. First, each model is trained and evaluated under grouped cross-validation by set_id so that no set contributes to both training and test partitions within a fold, and every data-dependent transformation is fit on the training portion only and applied unchanged to the held-out portion. Second, multimodality is introduced through explicit set-aligned feature blocks, assembled by merging process and engineered physics descriptors with pooled pore, grain, and stress–strain-derived descriptor blocks, with missingness tracked as a structural property of the dataset rather than treated as an error. Third, the key methodological distinction relative to many morphology-augmented surrogates is the separation between an oracle regime and a deployable regime: morphology proxies are treated as informative but incompletely observed, so modality embeddings are constructed within training folds and then used either as oracle embeddings when present or as predicted embeddings inferred from process features when morphology is absent. This design explicitly avoids reporting oracle-only improvements as deployable gains and introduces an embedding predictability diagnostic as a necessary condition for deployable multimodal inference. Finally, the surrogate stack is integrated into a decision layer that uses fold ensembles to produce prediction means and dispersion estimates and applies conservative lower-confidence bounds for candidate screening and ranking under constraints.

The results sought are to establish a deployable surrogate workflow that is stable under small sample size and partially observed modalities, and that supports downstream design decisions without assuming unavailable measurements. Concretely, the objectives are to obtain target-wise, fold-aggregated performance under the fixed GroupKFold protocol, to quantify the oracle versus deployable gap for morphology information, and to determine which modality embeddings are predictable from process variables with non-trivial fidelity using out-of-fold diagnostics. In addition, the study aims to produce a reproducible co-design procedure that generates a finite candidate pool within an observed process envelope, computes engineered physics features consistently, propagates predicted embedding when required, and evaluates feasibility and ranking under uncertainty-aware constraints using a fixed candidate budget.

The significance is that the work provides a deployment-aligned template for recipe-level learning and uncertainty-aware co-design in L-PBF Ti-6Al-4V, where conclusions depend on strict leakage control, explicit treatment of missing modalities, and separation of scientific upper bounds from decision-time feasibility [17,18]. The oracle versus deployable comparison yields an interpretable bound on the value of morphology information and clarifies when additional characterization is necessary versus when morphology can be treated as a latent state recoverable from process descriptors for prospective screening [19,20,21]. By incorporating uncertainty into constraint satisfaction and ranking, the framework reduces the risk of brittle recipe selection and supports multi-objective tuning for applications in which bulk mechanical requirements must coexist with surface-relevant constraints. Although developed for L-PBF Ti-6Al-4V, the present framework is transferable to other process–structure–property systems with sparse, partially observed multimodal data, provided that system-specific descriptors and target variables are reformulated and the models are retrained and prospectively validated in the new domain.

## 2. Materials, Data Sources, and Preprocessing

A recipe-level dataset for L-PBF Ti-6Al-4V is assembled such that each manufacturing condition is treated as a single independent set (set_id, 1–42), and all endpoints are represented at this set level using arithmetic means consistent with the database convention. Multimodal descriptors (process/engineered physics and morphology/microstructure proxies) are aligned to set_id, while non-uniform modality/label availability is treated as intrinsic and managed through explicit missing-data handling and grouped cross-validation with fold-local preprocessing to prevent leakage, as provided in Appendix A.

All analyses were conducted in a Conda-managed Python 3.11.15 environment on Windows, using NumPy 2.4.3, pandas 3.0.1, SciPy 1.17.1, scikit-learn 1.8.0, statsmodels 0.14.6, matplotlib 3.10.8, seaborn 0.13.2, openpyxl 3.1.5, and pyarrow 23.0.1.

**Figure 1:**
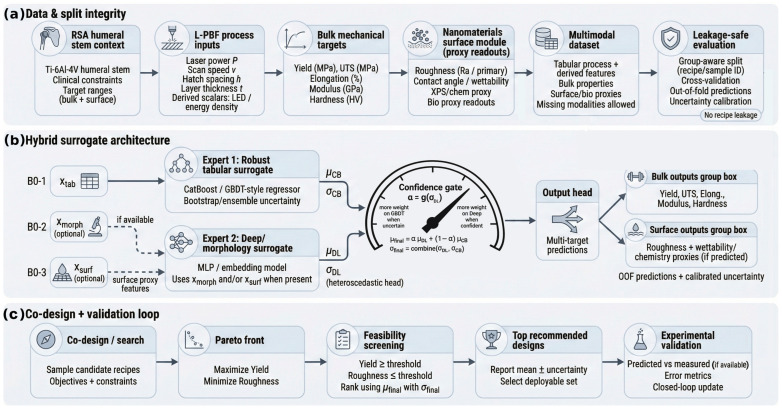
Deployment-aligned workflow for hybrid multimodal surrogate modeling and uncertainty-aware co-design in L-PBF Ti-6Al-4V. (**a**) Data construction and split-integrity pipeline linking recipe-level process inputs, bulk mechanical targets, and nanomaterials/surface proxy readouts in a multimodal dataset with missing modalities allowed under leakage-safe evaluation. (**b**) Hybrid surrogate architecture combining a robust tabular expert and an optional morphology/surface expert through a confidence-gated fusion module to produce calibrated multi-target predictions with uncertainty. (**c**) Co-design and validation loop for candidate generation, Pareto and feasibility screening, uncertainty-aware ranking, top-design selection, and experimental validation when measurements are available.

Figure 1 summarizes the leakage-safe data construction (Figure 1a) and provides a schematic of the deployable surrogate and uncertainty-aware co-design loop evaluated in later sections (Figure 1b,c).

### 2.1. Study Design and Set-Level Unit of Analysis (set_id)

The analytical unit is the set, indexed by set_id (range 1–42), where each set corresponds to a unique L-PBF manufacturing condition (recipe). All observations associated with the same set_id are treated as non-independent; this design prevents pseudoreplication arising from within-condition dependence when multiple specimens or repeated measurements exist for a given recipe.

**Figure 2:**
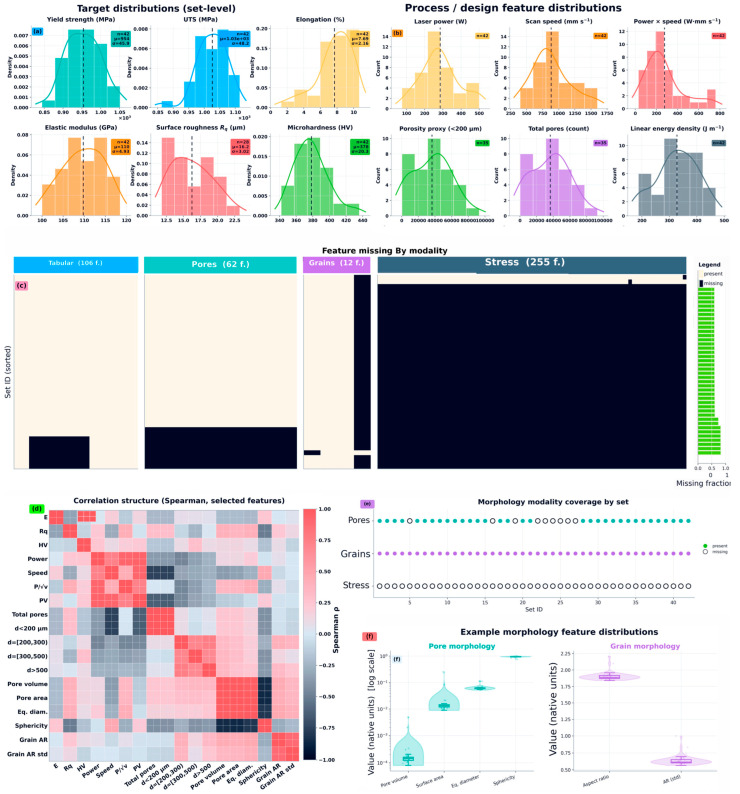
Dataset characterization for multimodal L-PBF Ti-6Al-4V surrogate modeling, with explicit separation between outputs and inputs. (**a**) Empirical distributions of the six output targets: yield strength, ultimate tensile strength, elongation, elastic modulus, surface roughness, and Vickers microhardness. (**b**) Representative input-feature distributions, including the primary raw process variables retained in the present dataset (laser power and scan speed), deterministic engineered descriptors derived from them (e.g., P×v, LED), and representative proxy features used as model inputs. (**c**) Feature-block missingness by modality (tabular/process, pores, grains, stress–strain). (**d**) Spearman rank-correlation matrix spanning outputs and selected input features. (**e**) Modality availability across set_id, highlighting non-uniform proxy coverage. (**f**) Representative pore- and grain-morphology feature distributions (shown on appropriate scales) to illustrate within-dataset variability and heavy-tailed behavior.

All endpoints are represented at the set level. Mechanical and surface responses are collapsed to a single set-level target value using the arithmetic mean consistent with the database convention (suffix “__mean”; target aggregation defined in Section 2.3). Target completeness differs across endpoints, with roughness exhibiting partial label availability relative to the mechanical properties, motivating an explicit missing-data policy and fold construction that preserve the set_id grouping (Section 2.5 and Section 2.6; Figure 2c).

Multimodal descriptors are aligned to set_id prior to modeling. The feature space comprises (i) process inputs and engineered physics features and (ii) morphology and microstructure proxies derived from object-level pore and prior-β grain descriptors and, when available, stress–strain-derived descriptors. Within-set heterogeneity is not discarded; object-level distributions are represented through pooled moments and quantiles to retain dispersion and tail behavior in the input space (Section 2.4; Figure 2e,f). Leakage control is implemented procedurally by grouped cross-validation, where train/test splits are performed by set_id so that no set contributes to both training and evaluation within a fold (Section 2.6).

### 2.2. L-PBF Process Parameter Space and Engineered Physics Features

The primary covariates include L-PBF process parameters defining the processing window (for example, laser power P, scan speed v, hatch spacing h, and layer thickness t, where available). To provide compact, physically motivated representations of the process space, energy-density variants are constructed in addition to the raw parameters. In particular, the line energy density (LED) is defined as

(1) $$LED=\;\frac{P}{v},$$

where P denotes laser power, and v denotes scan speed. When geometric terms are available, a volumetric energy-density variant consistent with standard L-PBF practice is included,

(2) $$VED=\;\frac{P}{v\;h\;t},$$

where h is hatch spacing, and t is layer thickness. These descriptors are treated as nominal energy-input proxies that support comparisons across parameterizations and complement the base process variables; their inclusion alongside constituent parameters is an expected redundancy within the locked feature specification rather than a post hoc feature-selection choice. The resulting operating-space coverage and the distributional support of key variables are reported in Figure 2b, and the overall block structure of inputs feeding the surrogate models is summarized in Figure 2a. The conditional availability of h and t is handled under the missingness and feature-filtering policy described in Section 2.5; feature definitions and units are given in Appendix A.3.

In Figure 2, we explicitly distinguish output targets from model inputs. Among the inputs, laser power and scan speed are the primary raw controllable process variables visualized in the present dataset, whereas quantities such as P×v and LED are deterministic engineered descriptors derived from those variables and are included as supplementary covariates rather than as independent process controls. Pore-related quantities shown in Figure 2 are likewise not treated as raw process parameters; they are morphology/proxy inputs derived from post hoc characterization and are analyzed separately at the modality level in Figure 2c–f.

### 2.3. Target Definitions: Yield, UTS, Elongation, Modulus, Roughness, Hardness

Six set-level response variables quantify mechanical performance and surface quality: 0.2% offset yield strength (yield, MPa), ultimate tensile strength (UTS, MPa), total elongation to fracture (elongation, %), Young’s modulus (modulus, GPa), surface roughness (roughness, µm), and Vickers microhardness (hardness, HV). Each target is represented at the set level, consistent with the unit of analysis defined in Section 2.1, such that each row corresponds to a unique build condition and its associated measurement set. When replicate measurements or multiple measurement locations are available for a given set, the reported target is the within-set arithmetic mean, aligned with the database convention (suffix “__mean”) as defined in Equation (3),

(3) $${\bar{y}}_{s}=\frac{1}{n_{s}}\sum_{j=1}^{n_{s}}y_{s,j},$$

where y_s,j_ denotes the jth measurement for set s, and n_sis_ the number of measurements available for that set and target.

The empirical distributions of the six targets, including skew and outliers, are summarized in Figure 2c. Dataset-level completeness differs by target, with roughness exhibiting partial label availability relative to the mechanical properties; this motivates explicit missing-data handling and evaluation procedures described in Section 2.5 and Section 2.6. Target definitions and units, together with the number of labeled sets per target, are reported in Table 1.

### 2.4. Morphology and Microstructure Proxies

Morphology and microstructure information is available as object-level measurements (for example, individual pore or grain objects) that vary in count across builds and imaging fields. To enable consistent learning at the set level, all morphology modalities are reduced to fixed-length set-level descriptors by statistical pooling over the object-level distributions within each set_id. This produces a tabular representation that can be merged with the L-PBF process variables and engineered physics features while preserving dominant distributional characteristics (central tendency, spread, tails, and extremes) relevant to defect populations and microstructural heterogeneity; modality-specific feature construction and aggregation rules are detailed in Appendix A.4 (Figure 2a,e).

Let $\{x_{i}{\}}_{i=1}^{n}$ denote an object-level scalar descriptor within one set, such as pore equivalent diameter, pore sphericity, pore volume, or grain aspect ratio, with nobjects detected for that set. The pooled set-level moments and quantiles are given in Equation (4).

(4) $$\mu =\frac{1}{n}\sum_{i=1}^{n}x_{i},\sigma =\sqrt{\frac{1}{n-1}\sum_{i=1}^{n}{\left(x_{i}-\mu \right)}^{2}},Q_{p}=\;{quantile}_{p}(\{x_{i}\}).$$

For each primitive descriptor, the pooled feature block includes {n, ∑, μ, σ, min, max, Q50, Q90, Q95, Q99}, together with derived transforms for heavy-tailed distributions (for example, ${log}_{10}(\cdot )$ variants of strictly positive measures). The resulting morphology features are therefore interpretable distribution summaries and can be directly analyzed for coverage and missingness (Figure 2e) and for redundancy against process and engineered features (Figure 2d). These pooled summaries also provide the input representation used by subsequent morphology-module experiments that construct low-dimensional morphology embeddings under fold-respecting procedures (Appendix C).

#### 2.4.1. Pore Morphology Summary Features

Pore morphology proxies summarize the size, shape, and population structure of defect pores within each set. Object-level pore descriptors include geometric measures such as volume, equivalent diameter, surface area, and sphericity; these are pooled per set using the statistics above, yielding a fixed set-level pore feature vector. Tail-sensitive summaries (high quantiles) are retained to reflect the process relevance of extreme pores. Where pore counts are low or absent for a set, the resulting set-level pore block is treated as partially observed rather than forced to zero, and missingness is handled under the policy in Section 2.5.

#### 2.4.2. Prior-β Grain Morphology Summary Features

Prior-β grain morphology proxies are constructed analogously from object-level grain measurements, capturing microstructural anisotropy and characteristic length scales. Representative descriptors include grain aspect ratio and equivalent diameter (or closely related size measures, depending on the raw measurement schema). These descriptors are pooled per set using the same moment and quantile statistics, forming a grain-summary block that is compatible with the unified set-level modeling table. Because grain measurements may be present for a subset of sets only, their availability is tracked explicitly and incorporated into missingness accounting (Figure 2e).

#### 2.4.3. Stress–Strain-Derived Descriptors

When stress–strain curves are available, they provide an additional source of morphology-adjacent proxy descriptors that can be used when direct imaging modalities are incomplete. In this case, each curve is reduced to a compact feature representation using physically interpretable summaries derived from the curve shape, such as slope-based stiffness proxies, characteristic stress and strain landmarks, and scalar descriptors that capture hardening behavior. These descriptors are treated as a separate modality block and incorporated at the set level under the same unified feature table, with their missingness recorded and handled using the procedures in Section 2.5 (Figure 2e).

More modest or inconsistent gains from morphology augmentation for certain targets should not be interpreted as evidence that the prior-β grain descriptors are irrelevant. Rather, the prior-β grain block captures only one part of the latent microstructural state. Stress–strain-derived descriptors integrate the cumulative effects of prior-β morphology together with pore population, α/α′ lath morphology, α/β phase constitution, retained β, texture, residual stress, and lattice-defect/dislocation structure. Accordingly, only partial correlations between grain summaries and stress–strain descriptors are expected, and the physically relevant signal is better interpreted at the modality/latent-embedding level than at the level of simple pairwise raw-feature correlation.

### 2.5. Missingness, Imputation Policy, and Feature Filtering

The compiled set-level table is multimodal and partially observed because modality availability is non-uniform across sets, with certain sets lacking pore, grain, or stress–strain descriptors depending on upstream data availability. Missingness is treated as an intrinsic property of the study design rather than an error condition. Missingness is quantified at both the feature level and the modality-block level to separate sporadic missingness from structurally absent modalities (Figure 2e). Targets are also incompletely observed for specific properties; modeling for a given target is performed only on the labeled subset for that property (Figure 2c).

To control dimensionality and stabilize learning under small-N conditions, feature filtering is applied prior to model fitting using a coverage threshold defined as a minimum required non-null fraction across sets (default criterion: at least 0.70 non-null coverage). This coverage filtering is performed at the feature-definition stage, and the resulting retained feature list is used consistently across subsequent modeling blocks to prevent feature drift and preserve comparability (Figure 2d,e). Constant or near-constant columns are additionally removed to avoid degenerate predictors.

For remaining missing values within retained features, imputation is performed using a fold-respecting rule in which imputation parameters are estimated on the training portion of each fold and applied to the corresponding validation or test portion. Median imputation is adopted as the default policy because it is robust under heavy-tailed distributions and small-sample settings and does not impose parametric assumptions that are rarely defensible in sparse multimodal materials datasets. Denoting the training-set median for feature j in fold k by ${\tilde{x}}_{j}^{\left(k\right)}$, missing values are imputed as

(5) $$x_{ij}\leftarrow \left\{\begin{array}{cc} x_{ij}, & \mathrm{if}\;x_{ij}\;\mathrm{is}\;\mathrm{observed},\\ {\tilde{x}}_{j}^{\left(k\right)}, & \mathrm{if}\;x_{ij}\;\mathrm{is}\;\mathrm{missing}. \end{array}\right.$$

When a model requires standardized inputs, standardization is likewise fit on the training portion of each fold only. With training mean $\mu_{j}^{\left(k\right)}$ and standard deviation $\sigma_{j}^{\left(k\right)}$, the standardized value is

(6) $$z_{ij}=\frac{x_{ij}-\mu_{j}^{\left(k\right)}}{\sigma_{j}^{\left(k\right)}},$$

with $\mu_{j}^{\left(k\right)}$ and $\sigma_{j}^{\left(k\right)}$ computed exclusively from the training split. This fold-conditional preprocessing prevents information leakage by disallowing test-fold statistics from influencing training-fold preprocessing; details are provided in Appendix A.5.

### 2.6. Train/Test Protocol: GroupKFold by set_id and Leakage Controls

Model evaluation follows a 5-fold grouped cross-validation protocol in which all rows sharing the same set_id are assigned to the same fold. This prevents optimistic bias caused by within-set correlations by ensuring that no set contributes to both training and test partitions within a fold. Let G denote the set of unique set_id groups. For fold k, the training and test group sets $G_{train}^{\left(k\right)}$ and $G_{test}^{\left(k\right)}$ satisfy Equation (7).

(7) $$G_{train}^{\left(k\right)}\cap G_{test}^{\left(k\right)}=\emptyset ,\overset{5}{\underset{k=1}{⋃}}G_{test}^{\left(k\right)}=G.$$

The normalized split file spans all 42 sets, with fold sizes of train/test = 33/9 for folds 1–2 and 34/8 for folds 3–5 (Figure 2f).

Leakage controls are enforced by performing every data-dependent operation strictly within each training fold and applying the learned transformation to the corresponding test fold; this includes imputation parameters, standardization parameters for scale-sensitive models, and any learned transformations used within the modeling pipeline. Hyperparameters are tuned via discrete grids using training-fold internal validation, and the selected best setting is recorded per fold and target.

**Figure 3:**
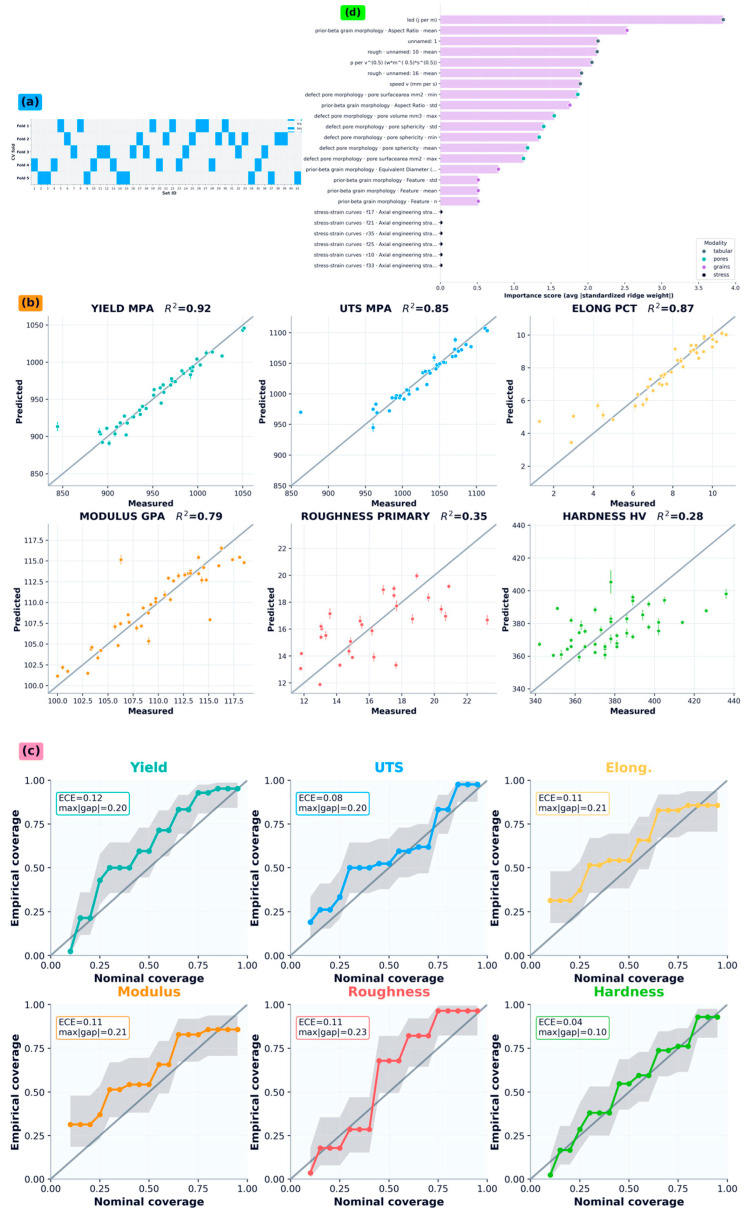
Model evaluation, uncertainty quantification, and interpretability under GroupKFold. (**a**) GroupKFold protocol showing test-fold assignment by set_id. (**b**) Out-of-fold parity plots for the six targets, with predictive uncertainty overlaid. (**c**) Split-conformal uncertainty calibration (GroupKFold-safe), reporting empirical versus nominal coverage with summary calibration metrics. (**d**) Global feature importance aggregated across folds, stratified by modality (process/tabular, pores, grains, stress–strain).

For targets with partial label availability, evaluation is performed on the labeled subset within each fold’s held-out partition. For roughness, 28 of 42 sets are labeled; folds contribute to the reported metrics through the labeled test instances only, avoiding artificial inflation of performance from label handling. Performance is reported as the mean and standard deviation across folds for each target using consistent metrics to enable direct comparison across models and modality variants (Figure 3a).

## 3. Surrogate Modeling Framework

Surrogate models are formulated to predict each set-level target using the leakage-resistant 5-fold GroupKFold protocol grouped by set_id (Section 2.6), with fold-local preprocessing for operations that depend on data statistics (Section 2.5); details are provided in Appendix B.1.

The modeling workflow proceeds from (i) tabular-only baselines using L-PBF process descriptors and engineered physics features to (ii) hybrid multimodal surrogates that append set-aligned morphology and proxy descriptor blocks (pores, prior-β grains, and stress–strain descriptors) under a consistent set-level unit of analysis. The locked modeling, preprocessing, and decision-layer settings adopted throughout the study are summarized in Table 2.

### 3.1. Baseline Tabular Models and CPU-Only Training Setup

Surrogate models are trained to predict each set-level target from the L-PBF process descriptors and engineered physics features defined in Section 2.2, evaluated under the GroupKFold protocol in Section 2.6. A suite of tabular regressors spanning linear, kernel, bagging, and boosted-tree families establishes a CPU-feasible reference for each property: ridge regression, k-nearest neighbors’ regression, RBF-kernel support vector regression, random forest regression, extremely randomized trees, histogram-based gradient boosting, and gradient-boosted decision trees; details are provided in Appendix B.2.

All experiments are executed under a CPU-only workflow on an Intel(R) Xeon(R) Silver 4214 CPU @ 2.20 GHz (no GPU acceleration). Models are refit from scratch within each fold. Targets are modeled independently (one regressor per property) due to differences in label availability and noise structure and to allow for target-specific model selection under the same evaluation protocol.

Preprocessing steps that use fold-dependent statistics (imputation and any scaling) are fit on the training portion only and applied unchanged to the held-out portion. Coverage-based feature filtering is applied at the feature-definition stage using the full set-level table to remove sparse or degenerate columns, while fold-wise imputation and scaling remain strictly confined to training-only statistics (Section 2.5). Baseline performance is reported as mean and standard deviation across folds; the best-performing baseline per target is identified under this fixed protocol and carried forward as the reference for later modality deltas (Table 3).

### 3.2. Hybrid Multimodal Feature Construction

Hybrid multimodal surrogates extend the tabular baseline by concatenating set-level morphology and proxy descriptors derived from additional modalities (Section 2.4). The unified design matrix is assembled by aligning and merging four set-level blocks by set_id: (i) tabular process and engineered physics descriptors, (ii) pore morphology summary features, (iii) prior-β grain morphology summary features, and (iv) stress–strain-derived descriptors (when available) retained as a proxy block; details are provided in Appendix A.6. Modality coverage and missingness are tracked explicitly (Figure 2e).

Because modality blocks differ in dimensionality and sparsity, hybrid construction applies controlled feature screening and fold-safe imputation. Candidate columns are screened by non-null coverage, and degenerate columns are removed; the remaining missing entries are imputed using fold-specific training medians (Section 2.5). Hybrid variants are constructed as modality ablations to isolate incremental contributions, including tabular-only, tabular + pores, tabular + grains, tabular + pores + grains, tabular + stress, and tabular + all-morphology. These variants are trained and evaluated under the same GroupKFold protocol as the tabular baselines, enabling direct comparisons under identical leakage controls and CPU constraints (Table 3; Figure 3a,b).

Two integration settings are supported within the same framework. In the direct-fusion setting, pooled morphology statistics are appended as explicit numeric features to enable cross-modal interactions in downstream learners. In the deployable two-stage setting, modality embeddings are learned within each training fold and then used as oracle embeddings when present or predicted from the tabular block when absent, separating the representational value of morphology from deployment-time availability constraints.

### 3.3. Hyperparameter Selection and Evaluation Metrics

Hyperparameter selection followed a leakage-controlled workflow consistent with the 5-fold GroupKFold protocol by set_id, such that candidate configurations were trained using training data only within each fold and the held-out fold was accessed only for final scoring.

All data-dependent operations that can transmit distributional information (including imputation, standardization, and any coverage-based filtering rules) were confined to training-fold computations and then applied unchanged to the corresponding held-out fold to preserve the leakage barrier.

Hyperparameters were tuned via discrete grids using training-fold internal validation, with the selected setting recorded per fold and per target to maintain auditability under small-N conditions; full tuning grids and statistical reporting details are given in Appendix B.2 and Appendix B.4.

Table 2 formalizes the invariant methodological assumptions adopted throughout the study, including the recipe-level unit of analysis (set_id), the grouped cross-validation design, and strictly fold-local preprocessing operations that preclude information leakage. By locking these protocol elements across all model families, the empirical comparisons reported in subsequent sections isolate the effect of the surrogate and uncertainty modules from confounding variation in data handling. In addition, the morphology and co-design components are treated as version-controlled, archived configurations, ensuring that both predictive evaluations and downstream design recommendations are reproducible under an identical experimental specification. For boosted-tree libraries, early stopping was used only when supported by the installed package API; otherwise, a fixed estimator budget was used and model selection proceeded through the declared discrete grid to preserve version-robust reproducibility. Evaluation was reported using complementary error and goodness-of-fit metrics to capture both absolute deviation and explained variance across targets with different scales. Root mean squared error (RMSE) was used as the primary scale-dependent metric due to its sensitivity to large deviations, mean absolute error (MAE) was reported as a robust companion metric, and the coefficient of determination (R^2^) was reported relative to a constant-predictor baseline.

Using targets $y_{i}$ and predictions ${\hat{y}}_{i}$ over n test instances, the metrics are

(8) $$RMSE=\sqrt{\frac{1}{n}\sum_{i=1}^{n}{\left(y_{i}-{\hat{y}}_{i}\right)}^{2}},$$

(9) $$MAE=\frac{1}{n}\sum_{i=1}^{n}|y_{i}-{\hat{y}}_{i}|,$$

(10) $$R^{2}=1-\frac{\sum_{i=1}^{n}{\left(y_{i}-{\hat{y}}_{i}\right)}^{2}}{\sum_{i=1}^{n}(y_{i}-\bar{y}{)}^{2}},$$

(11) $$\bar{y}=\frac{1}{n}\sum_{i=1}^{n}y_{i}.$$

All metrics were computed on the held-out fold and aggregated across the five folds as mean ± standard deviation to capture both expected performance and partition sensitivity in the small-N regime.

For the roughness target, only 28 of 42 sets are labeled; evaluation therefore uses labeled instances in each fold’s held-out partition only, and folds contribute to reported metrics through those labeled test instances.

The main comparative results are summarized in Table 3; model settings and the locked feature schema are documented in Table 2 to diagnose systematic bias and heteroscedastic error patterns not visible in scalar metrics alone.

### 3.4. Uncertainty Estimation Strategy

Predictive uncertainty was estimated using fold ensembles induced by the GroupKFold partitions. For each target, one model per fold was trained using the selected architecture and target-specific feature variant; at inference, each input yields an ensemble of fold-trained predictions $\{{\hat{y}}^{\left(k\right)}(x){\}}_{k=1}^{K}$ with K=5.

The predictive mean was defined as the ensemble mean,

(12) $$\mu (x)=\frac{1}{K}\sum_{k=1}^{K}{\hat{y}}^{\left(k\right)}(x),$$

and epistemic spread was summarized using the ensemble standard deviation,

(13) $$\sigma (x)=\sqrt{\frac{1}{K-1}\sum_{k=1}^{K}{\left({\hat{y}}^{\left(k\right)}(x)-\mu (x)\right)}^{2}}.$$

These summaries are deployable in the sense that they require only the trained fold models and do not assume access to additional calibration labels at inference time.

Uncertainty was used in two roles: (i) surrogate-level diagnostics via coverage-style calibration checks (Figure 3b), where predicted uncertainty is assessed against empirical error behavior across nominal quantiles, and (ii) decision-making in co-design to penalize overly optimistic candidates through a conservative lower-confidence bound (LCB) in Equation (14).

(14) LCB(x)=μ(x)−z σ(x),

where z controls conservativeness.

The co-design recommendation table (Table 4) reports mean predictions and uncertainty summaries to keep the ranking and screening logic transparent and auditable.

### 3.5. Optimizer Ablation Protocol

An optimizer ablation quantified sensitivity of training dynamics and generalization performance to optimizer choice under an identical model architecture, identical feature set, and the same 5-fold GroupKFold protocol by set_id, so that observed differences reflect optimization behavior rather than data partition effects. The study compared AdamW, Muon, and a hybrid strategy in which Muon was applied selectively to a subset of parameters (hidden layers) while AdamW was retained for remaining parameter groups; additional optimizer-ablation settings, learning-rate sweeps, and selection rules are detailed in Appendix B.3.

The optimizer study was framed as a robustness evaluation for a fixed neural tabular regressor, with identical splits and identical preprocessing across optimizers to isolate optimizer effects. For each target property, training was repeated across multiple random seeds for each optimizer to separate optimizer-induced variance from partition-induced variance. Within each fold, each optimizer used the same initialization scheme, stopping criterion, and maximum training budget; performance was recorded on the held-out fold using RMSE as the primary metric, with MAE and R^2^ tracked as secondary diagnostics.

Learning-rate operating regions were characterized via grid sweeps for each optimizer. A discrete set of learning rates was evaluated for each target, and the best-performing learning rate was selected based on fold-aggregated validation performance, subject to stability screening (excluding configurations that diverged or produced degenerate predictions).

**Figure 4:**
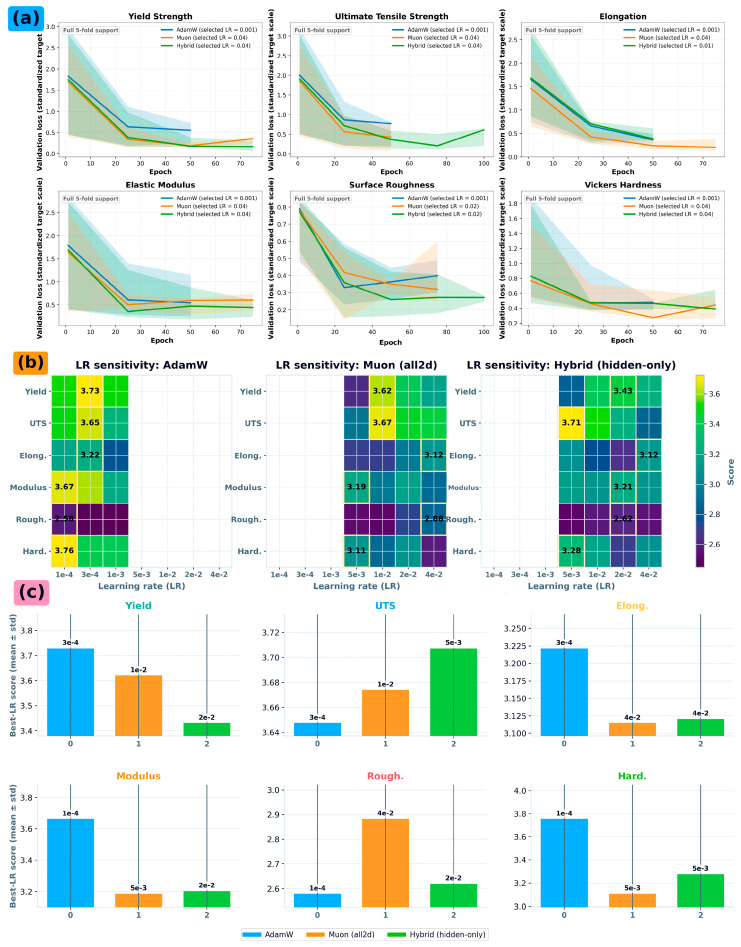
Optimizer stability and learning-rate sensitivity for the neural tabular regressor under the fixed 5-fold GroupKFold protocol grouped by set_id. (**a**) Fold-aggregated validation trajectories at the selected learning-rate operating point for each optimizer and target. Solid lines show the median validation loss across folds, and shaded bands show the interquartile range. Only epochs with full 5-fold support are shown to avoid overinterpreting late-stage variance after early stopping. (**b**) Learning-rate operating regions in score space for each optimizer across the six targets, showing the range of stable and competitive learning-rate choices. (**c**) Best learning-rate operating point per optimizer and target, reported as fold-aggregated mean performance with variability, with labels indicating the selected learning rate.

The resulting score landscapes were summarized, best achievable operating points per optimizer and target were summarized as mean ± standard deviation across runs, and run-to-run dispersion at the selected learning rate was reported via boxplots (Figure 4a). To avoid confounding from feature-definition differences, the optimizer ablation used a locked feature schema and a fixed preprocessing pipeline, with median imputation and any scaling computed on training-only partitions and applied with frozen parameters to validation/test partitions following the leakage controls in Section 2.5 and Section 2.6.

## 4. Morphology Module: Two-Stage Embedding and Deployable Multimodal Inference

Morphology and microstructure proxies (pore statistics, prior-β grain descriptors, and stress–strain-derived signatures) carry mechanistically relevant information, yet their availability is non-uniform across sets, creating a fundamental train-time-versus-deploy-time information mismatch. A two-stage morphology module is therefore used to separate an oracle regime, in which proxies are observed at inference, from a deployable regime, in which low-dimensional proxy embeddings are learned within training folds and then predicted from process-derived inputs when proxies are absent.

### 4.1. Oracle Morphology vs. Predicted Morphology Concept

Morphology and microstructure proxies, including pore morphology, prior-β grain morphology, and stress–strain-derived descriptors, are informative but incompletely observed across sets. This creates a practical distinction between a train-time setting in which proxy descriptors are available for supervised learning and a deploy-time setting in which these descriptors may be absent for a new candidate process recipe. The morphology module is therefore formulated as a two-stage mechanism that separates (i) the informational value of morphology-conditioned inference when proxy descriptors are observed from (ii) the feasibility of producing morphology-conditioned predictions when proxy descriptors are missing.

Two inference regimes are considered. In the oracle regime, proxy descriptors are treated as directly observed inputs at inference time. This regime estimates an upper bound on achievable surrogate performance when multimodal measurements are available and is used to quantify the potential benefit of morphology-aware learning. In the deployable regime, proxy descriptors are not assumed to be observed for candidate designs. Instead, proxy embeddings are first predicted from the available tabular/process features, and the predicted embeddings are then used as substitutes for the missing proxy inputs in the final property surrogate. This yields a deployable multimodal inference path that can be applied to co-design candidate generation, where only process parameters and engineered physics features are available.

Formally, let x denote the tabular/process feature vector (including engineered physics features), and let m denote a modality-specific proxy feature block (pores, grains, or stress). The oracle surrogate is defined in Equation (15).

(15) y=f([x,m]),

where $\left[\cdot ,\cdot \right]$ denotes concatenation. The deployable two-stage surrogate is defined by Equations (16) and (17).

(16) z=g(x),

and a property surrogate predicts the target using the predicted embedding,

(17) y=f([x,z]).

This separation makes explicit the train-time versus deploy-time information structure and avoids reporting oracle-only gains as deployable improvements. The comparison between oracle and deployable variants is summarized in the morphology-module results table and is used downstream to select target-specific deployable variants for co-design.

### 4.2. PCA Embedding Construction on Training Folds

Raw proxy feature blocks are high-dimensional and heterogeneous across modalities (for example, tens of pore summary features, a smaller set of grain descriptors, and a larger stress–strain descriptor set). To obtain compact representations that are easier to predict from tabular inputs and less prone to overfitting under small-sample conditions, each proxy modality is mapped to a low-dimensional embedding using principal component analysis (PCA). PCA fits strictly within each training fold to preserve leakage control, and the learned transformation is then applied to the corresponding held-out fold.

For each modality, a training-fold morphology matrix is constructed by selecting the modality-specific feature columns, coercing to numeric types, and applying training-only imputation. Let $M_{train}^{\left(k\right)}\in R^{n_{k}\times d}$ denote the training-fold matrix for fold k. PCA is fit to $M_{train}^{\left(k\right)}$, and the number of retained components q is chosen using a cumulative explained-variance criterion with an upper cap to avoid excessive flexibility under small n. Specifically, the smallest q is selected such that cumulative explained variance exceeds 95%, subject to $q\leq q_{max}$. The resulting training-fold embedding is

(18) $$Z_{train}^{\left(k\right)}={PCA}^{\left(k\right)}\;(M_{train}^{\left(k\right)}),$$

and the held-out embedding is obtained by applying the same transformation to the test-fold matrix $M_{test}^{\left(k\right)}$,

(19) $$Z_{test}^{\left(k\right)}={PCA}^{\left(k\right)}\;(M_{test}^{\left(k\right)}).$$

This fold-specific fitting ensures that no distributional information from held-out sets influences the embedding basis. Because proxy availability differs by modality and by set, modality-specific usability checks are applied per fold. If a modality has insufficient observed values or degenerate variance after preprocessing in each fold, the modality embedding is treated as unavailable for that fold to avoid unstable PCA solutions. The final embedding dimensionalities used in experiments are therefore reported explicitly (per modality) alongside the downstream property-surrogate results. The PCA embeddings constitute the interface between proxy observation and deployable inference; they serve as the targets for morphology prediction in the deployable pipeline and as conditioning variables for the oracle pipeline. A necessary condition for deployable multimodal inference is that morphology embeddings can be estimated from the available process-derived feature set with non-trivial fidelity [22,23,24]. Embedding predictability is therefore quantified directly by training fold-respecting predictors from tabular/process features to each PCA embedding coordinate and reporting out-of-fold values. This diagnostic is reported at the embedding level, not only at the downstream property level, because a two-stage pipeline can fail either due to weak embedding predictability or due to limited incremental utility of morphology even when predicted accurately.

Let $x_{s}$ denote the tabular/process feature vector for set s, and let $z_{s}^{\left(m,,,k\right)}\in R^{q_{m}^{\left(k\right)}}$ denote the PCA embedding for morphology modality m constructed within training fold k (Section 4.2), with fold-specific PCA fit on the training partition only and then applied to the held-out fold. Because PCA fits within each fold, embedding coordinates are fold-local; coordinate-wise predictability is interpreted as a within-fold diagnostic rather than a globally fixed latent axis.

For each fold k, a predictor $g_{m}^{\left(k\right)}$ is trained on $\left\{(x_{s},z_{s}^{\left(m,,,k\right)}):s\in G_{\mathrm{train}}^{\left(k\right)}\right\}$ and evaluated on $G_{\mathrm{test}}^{\left(k\right)}$. Predictability is summarized using the coefficient of determination,

(20) $$R_{z,j}^{2}(m,k)=1-\frac{\sum_{s\in G_{\mathrm{test}}^{\left(k\right)}}{\left(z_{s,j}^{\left(m,,,k\right)}---{\hat{z}}_{s,j}^{\left(m,,,k\right)}\right)}^{2}}{\sum_{s\in G_{\mathrm{test}}^{\left(k\right)}}{\left(z_{s,j}^{\left(m,,,k\right)}---{\bar{z}}_{j}^{\left(m,,,k\right)}\right)}^{2}},$$

**Figure 5:**
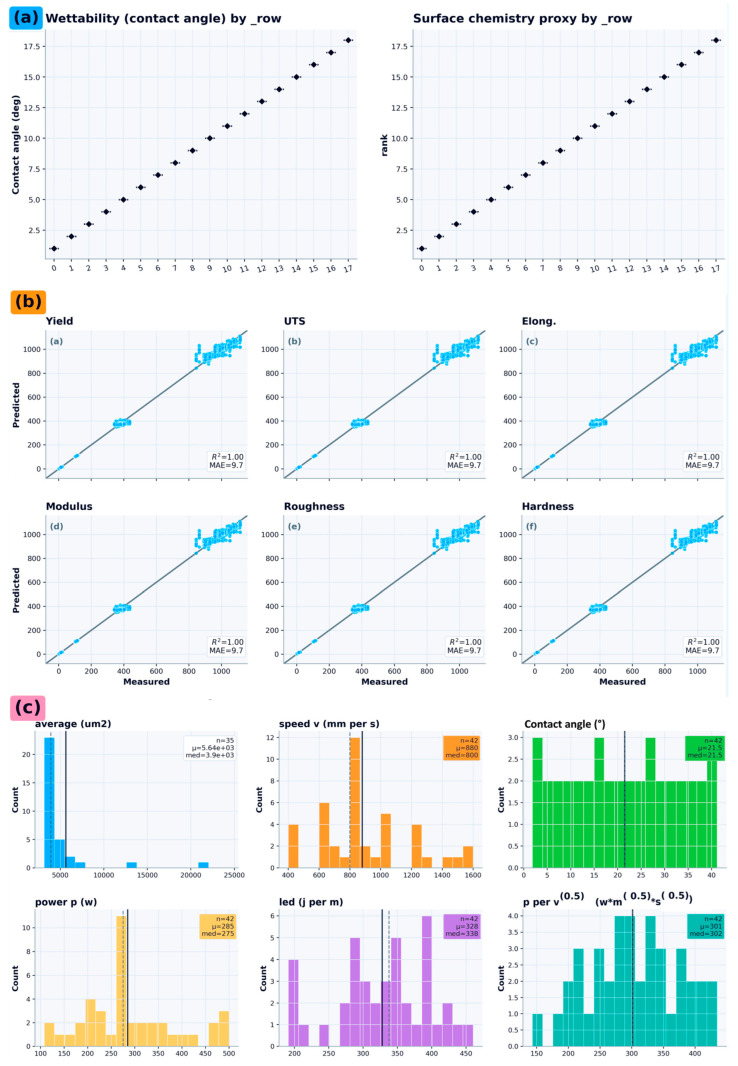
Surface-relevant proxy measurements and surrogate performance. (**a**) Wettability (contact angle) and a surface-chemistry proxy reported by row, illustrating monotonic ordering across experimental runs. (**b**) Parity plots for the surface surrogate across the six targets, summarizing predictive fidelity under the same evaluation protocol. (**c**) Distributions of bio- and surface-proxy readouts alongside key process/physics descriptors (e.g., speed, power, LED), highlighting the dynamic range and coverage used for downstream screening.

These diagnostics enable modality-wise screening for deployable inference; modalities with consistently low embedding predictability indicate limited learnability of the corresponding embeddings from the available process covariates under the present dataset and coverage, implying that oracle-only improvements should not be interpreted as deployable gains. This diagnostic is referenced in the morphology-module results (Figure 5, morphology module panel(a)) and in the selection of deployable variants used for co-design (Section 4.4).

### 4.3. Deployable Two-Stage Pipeline Used in Co-Design

The final co-design workflow requires property prediction for candidate process recipes before any physical build, which precludes direct observation of pores, grains, or stress–strain descriptors [25,26,27]. The deployable surrogate therefore uses a two-stage inference pathway in which morphology information is represented by predicted PCA embeddings derived from process variables.

#### 4.3.1. First-Stage Embedding Prediction

For each morphology modality selected for a given target, a modality-specific predictor is trained to map tabular/process features to the modality embedding,

(21) $${\hat{z}}_{s}^{\left(m,,,k\right)}=g_{m}^{\left(k\right)}(x_{s}),$$

with $g_{m}^{\left(k\right)}$ trained under the same GroupKFold protocol as the property surrogates. Training-only preprocessing is applied within each fold, including median imputation and any scaling required by the chosen model class (Section 2.5). Importantly, the PCA used to define $z^{\left(m,,,k\right)}$ is fit on the training partition only (Section 4.2), so the embedding predictor does not access held-out sets beyond strict cross-validation allowances.

#### 4.3.2. Second-Stage Property Prediction

The predicted embeddings are concatenated with the tabular/process features to form the deployable multimodal input, and a target-specific surrogate is trained to predict the mechanical or surface property of interest,

(22) $${\hat{y}}_{s}^{\left(k\right)}=\;f^{\left(k\right)}\;\left(\left[\;x_{s},\;{\hat{z}}_{s}^{\left(m_{1},,,k\right)},\ldots ,{\hat{z}}_{s}^{\left(m_{M},,,k\right)}\;\right]\right).$$

Model selection at this stage is target-specific and constrained to deployable variants, meaning that oracle-only inputs are excluded when choosing the final surrogate used in design-space exploration. When multiple modality combinations are feasible, the best deployable variant is selected per target based on mean cross-validated RMSE (and associated $R^{2}$), with the corresponding configuration recorded for reproducibility.

### 4.4. Uncertainty Summaries for Design-Time Ranking

**Figure 6:**
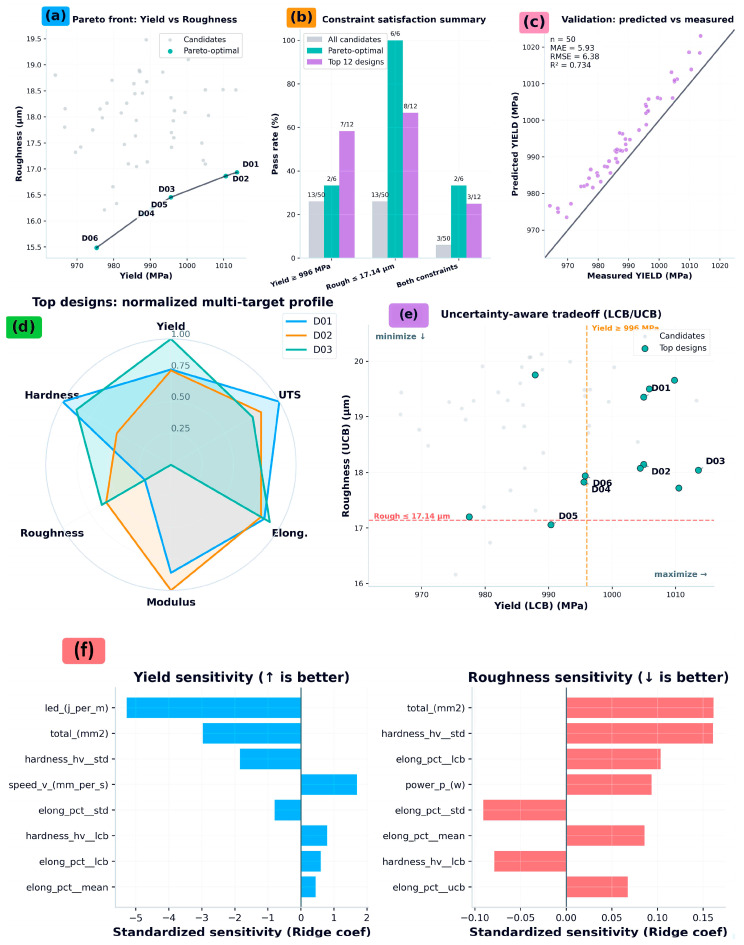
Co-design under constraints and uncertainty. (**a**) Pareto front for yield versus roughness, highlighting Pareto-optimal and selected designs. (**b**) Constraint-satisfaction rates for all candidates, Pareto-optimal designs, and the top-ranked subset. (**c**) Validation (predicted versus measured) for designs with measurements, with summary error metrics. (**d**) Normalized multi-target profiles for the top designs. (**e**) Risk-aware trade-off using confidence bounds (LCB/UCB) for yield and roughness. (**f**) Local process-parameter sensitivity around the candidate set, indicating robustness and dominant drivers.

Co-design requires not only point predictions but also a notion of predictive uncertainty to avoid brittle recommendations. For each candidate, the ensemble of fold-trained surrogates produces a distribution of predictions; the empirical mean serves as the primary estimate and the empirical standard deviation across the fold models serves as a conservative uncertainty proxy. These summaries are used to implement risk-aware selection and constraint handling in candidate screening, where feasibility is evaluated under uncertainty-aware criteria rather than single-point estimates. This deployable two-stage surrogate is used to generate the final recommendation table for co-design (Table 4) and to support the Pareto and risk-aware analyses in the co-design section (Figure 6).

## 5. Results

Performance evidence is organized to characterize the multimodal set-level learning problem, establish leakage-controlled baselines, and evaluate the deployable value of morphology augmentation through oracle versus predicted embeddings, culminating in uncertainty-aware co-design recommendations.

### 5.1. Dataset and Feature Characterization

Table 1 summarizes the final set-level dataset used for model development, including the unique set_id groups (1–42), GroupKFold fold membership, and target-specific label availability. The dataset is intrinsically multimodal at the set level, combining (i) L-PBF process parameters and engineered physics features and (ii) morphology and microstructure proxies derived from pore statistics, prior-β grain morphology, and stress–strain descriptors where available.

Figure 2 provides a compact characterization of the learning problem. Figure 2a depicts the set-level unit of analysis and the partitioning of inputs into process/physics features and morphology-derived blocks alongside the six targets. Figure 2b visualizes the coverage of the process parameter space and engineered energy-input descriptors, documenting the observed operating ranges represented in the set-level table. Figure 2c reports the empirical target distributions (yield strength, ultimate tensile strength, elongation, elastic modulus, surface roughness, microhardness), highlighting differences in scale, dispersion, and tail behavior that motivate target-wise treatment and inform interpretation of error magnitudes.

Figure 2 provides a compact characterization of the learning problem with explicit separation between outputs and inputs. Figure 2a shows the empirical distributions of the six set-level response variables. Figure 2b summarizes representative input-feature distributions; importantly, this panel combines the raw process variables retained in the present dataset with deterministic engineered descriptors derived from them and selected proxy features used by the multimodal surrogate. Figure 2c–f then characterize the input space further through modality-wise missingness, output–input correlation structure, modality coverage across set_id, and representative morphology-feature distributions. This organization is intended to distinguish clearly between the predicted outputs and the heterogeneous input blocks used in the surrogate framework.

Target completeness differs by endpoint; in particular, roughness has partial label availability relative to mechanical properties, and labeled-subset evaluation is used for targets with incomplete labeling. This target-dependent effective sample size is a dataset property that must be carried forward when comparing results across targets.

### 5.2. Baseline Performance Comparison

Baseline regressors are evaluated under the fixed 5-fold GroupKFold protocol to establish a CPU-only reference for each target. Table 3 reports fold-aggregated performance (mean ± standard deviation) using RMSE as the primary metric, with MAE and R^2^ reported as complementary diagnostics, and records the best-performing baseline per target under this protocol. Across the six targets, baseline results indicate that strong nonparametric learners provide competitive accuracy under small-n conditions, while target difficulty differs substantially. In particular, the baselines exhibit robust performance for strength and ductility targets (yield strength, UTS, elongation) and elastic modulus, whereas surface roughness and microhardness show weaker baseline performance.

This contrast is consistent with the combination of target distributional characteristics and the limited observability of morphology-dependent information in tabular-only features, especially under partial modality coverage. For partially labeled targets, reported headline values are computed across the subset of folds that contribute labeled test instances, and this reporting convention is maintained to preserve auditability under target-dependent completeness.

Table 3 provides the target-wise, fold-aggregated error statistics computed under the fixed 5-fold GroupKFold protocol, thereby enabling direct comparison across endpoints under a common leakage-controlled evaluation design. Consistent with target-dependent completeness, partially labeled endpoints are evaluated on the labeled subset of held-out sets rather than by imputing labels, preserving the auditability of reported metrics. Under this protocol, strength and ductility targets exhibit substantially higher explained variance than surface roughness and hardness, motivating subsequent sections that test whether additional morphology/proxy information can produce systematic gains beyond process/physics descriptors alone.

The baseline outcomes motivate the subsequent multimodal modeling stages, which incorporate morphology and microstructure proxies to test for systematic improvements beyond tabular process descriptors alone (Section 5.3, Section 5.4 and Section 5.5; Figure 3 and Figure 5; Table 3 and Table 4).

### 5.3. Final Hybrid Surrogate Performance, Parity Plots, and Global Feature Importance

Table 2 documents the final surrogate family and locked training settings used for the reported models, including the CPU-only implementation, the feature-construction variant adopted per target, and the final hyperparameter choices used in cross-validation.

Table 3 reports the corresponding predictive performance (RMSE, MAE, and R^2^) aggregated across the five GroupKFold splits, enabling direct comparison to the tabular-only baselines under an identical evaluation protocol.

Figure 3 consolidates the primary performance and interpretability evidence for the final hybrid surrogate. Figure 3a documents the GroupKFold evaluation protocol and the placement of model selection within each training fold, while Figure 3a summarizes target-wise performance and fold-to-fold spread using the same metric definitions reported in Table 3. This organization supports a direct assessment of whether incorporating morphology-derived information yields target-specific gains beyond process/physics features alone, rather than attributing improvements to procedural differences. Parity plots in Figure 3 provide an essential diagnostic complement to scalar metrics in the small-n regime. Predicted values are plotted against measured values for held-out folds, with the identity line used to assess systematic bias. The fold-aggregated error statistics shown in the panel insets correspond to the values tabulated in Table 3.

This parity-based view supports inspection of regime-dependent error structure (including outliers and tail behavior evident in the target distributions) that can be obscured by aggregate RMSE alone, and it provides a fold-respecting check against overfitting through consistency of held-out behavior; additional residual-based, modality-sensitivity, and robustness checks are provided in Appendix B.5. Global feature attribution for the final surrogate is summarized in Figure 3c. The ranked importances are drawn from the final gradient-boosted model family and each feature is associated with its modality block (process/physics versus morphology-derived descriptors).

This analysis is used as a mechanistic plausibility audit verifying that physically motivated energy-input descriptors and morphology summaries appear among dominant drivers, while remaining explicitly non-causal in interpretation. The feature-importance view is therefore treated as a global diagnostic to contextualize hybrid performance and to motivate the morphology-module analyses that follow.

### 5.4. Optimizer Stability and Learning Dynamics

Figure 4 evaluates the effect of optimizer choice on training stability and convergence behavior in the low-data regime, using the fixed experimental protocol defined in Section 3.5.

The primary objective of this analysis is robustness: determining whether reported surrogate accuracy is attributable to modeling choices rather than idiosyncratic training dynamics. Figure 4a reports fold-aggregated validation trajectories at the selected operating point for each optimizer and target, shown as the median validation loss across GroupKFold splits with interquartile bands. To avoid overinterpreting late-epoch variance after early stopping, only epochs with full 5-fold support are displayed. Under this aggregation, the dominant reduction in validation loss occurs during the early training phase for all targets, while the remaining late-stage variability is modest and is more pronounced for UTS and elongation than for yield. This pattern should not be interpreted as evidence of a distinct second convergence phase. Rather, it is consistent with the greater sensitivity of UTS and elongation to partially observed post-yield microstructural state variables, including α/β phase balance, α/α′ lath morphology, retained β, texture, grain-boundary α, residual stress, and lattice-defect/dislocation state. Accordingly, optimizer selection is based primarily on the fold-aggregated learning-rate operating regions and best-operating-point summaries in Figure 4b,c, rather than on the terminal value of any single trajectory.

Figure 4a reports stability distributions across repeated runs as boxplots of the validation metric for each optimizer variant, directly diagnosing sensitivity to initialization and run-to-run variability. Learning-rate operating regions are mapped as target-by-learning-rate heatmaps for each optimizer, identifying stable windows and revealing whether tuning tolerance is unusually narrow.

Best operating points are summarized for each optimizer–target pair by reporting the top mean performance with its variability, annotated with the corresponding selected learning rate. The optimizer ablation is used as a sensitivity analysis supporting reproducibility of the reported surrogate results and as justification for the final default optimizer and learning-rate setting adopted in Table 2.

Consistent with the broader leakage controls, the optimizer study is conducted on a locked feature schema and fixed preprocessing pipeline, with median imputation and any required scaling computed on training-only partitions and applied to held-out partitions using frozen parameters.

### 5.5. Morphology Module Outcomes: Oracle vs. Predicted Embeddings and Their Impact

Figure 5 reports the outcomes of the two-stage morphology module designed to separate training-time information from deploy-time feasibility. The central comparison is between oracle morphology embeddings, where morphology descriptors are computed directly from measured pore and grain summaries and then embedded within each training fold, and predicted morphology embeddings, where the same embedding coordinates are inferred from process and engineered physics features alone. This distinction is critical for deployment, because morphology measurements are not guaranteed to be available for prospective designs, while process parameters and engineered physics features are always available.

The morphology module and the two-stage training logic are introduced, clearly distinguishing the oracle pathway from the deployable predicted pathway. Figure 5a reports the embedding predictability diagnostics, quantified by fold-wise R^2^ between oracle embedding coordinates and their process-predicted counterparts; this panel establishes which morphology modalities can be reconstructed with meaningful fidelity from process variables and which remain weakly predictable. Figure 5b evaluates the downstream impact on property prediction by comparing tabular-only surrogates to hybrid surrogates augmented with oracle embeddings and with predicted embeddings, using the same GroupKFold protocol as in Figure 3. Improvements attributable to oracle embeddings bound the maximum achievable gain from morphology information under perfect availability, while improvements from predicted embeddings quantify what is realizable under deployable conditions. Figure 5c summarizes the resulting target-dependent pattern, highlighting cases where morphology information adds measurable value and cases where performance is dominated by tabular process and engineered features.

### 5.6. Co-Design Recommendations Under Constraints and Uncertainty

Table 4 reports the constrained co-design recommendations produced by the deployable surrogate stack, including the selected process parameter sets, predicted property means, and uncertainty summaries used for risk-aware ranking. Recommendations are generated from a candidate pool sampled within the observed process envelope and evaluated using fold ensembles to obtain both central tendency and dispersion estimates for each target. Constraint handling is performed by screening candidates against minimum performance thresholds and by ranking feasible solutions with uncertainty-aware criteria, ensuring that reported designs reflect both expected performance and predictive confidence.

Predicted targets are reported as fold-ensemble mean ± standard deviation (GroupKFold-safe fold ensembles). Yield LCB and Roughness UCB correspond to the conservative confidence bounds used in the uncertainty-aware selection analysis. Designs are ordered by the composite score used for ranking feasible candidates.

Figure 6 visualizes the co-design outcomes and the logic of recommendation selection. Figure 6a presents the Pareto front for the primary tradeoff axis, showing the candidate cloud, Pareto-optimal subset, and highlighted top recommendations. Figure 6b reports constraint satisfaction rates, comparing all candidates to Pareto-optimal and top-ranked subsets to demonstrate the tightening effect of constraints and ranking. Figure 6c provides the validation view where measured outcomes are available, plotting predicted versus measured performance for the evaluated designs with identity reference and summary metrics. Figure 6d summarizes the recommended recipe set in an interpretable parameter-space view, enabling rapid comparison of power, scan speed, hatch spacing, layer thickness, and derived energy descriptors across top candidates. Figure 6e reports the risk-aware selection analysis, contrasting mean performance with uncertainty or lower-confidence bounds to justify the final recommended subset. Figure 6f presents a local robustness analysis around the selected recipe, quantifying sensitivity to small perturbations in key process variables and indicating whether recommendations lie in locally stable regions rather than fragile optima.

## 6. Discussion, Conclusions and Data Availability

Interpretation is cast in a hierarchical process–structure–property paradigm, in which process settings and engineered energy-input descriptors index thermal history, with defect and microstructure proxies mediating the observed mechanical and surface responses [28,29,30].

The oracle–deployable comparison is used to delineate morphology information that is recoverable from process variables from irreducible signal that warrants targeted characterization, while uncertainty-aware selection is emphasized to avoid brittle optima under sparse and heterogeneous measurement coverage.

### 6.1. Materials-Science Interpretation of Dominant Drivers

The results are consistent with a hierarchical process–structure–property framing in which L-PBF process settings and engineered energy-input descriptors act as first-order proxies for the thermal histories that shape defect formation and microstructural evolution, which then mediate macroscopic properties. Within this interpretation, controllable inputs (laser power, scan speed, hatch spacing, layer thickness) together with linear energy density and volumetric energy-density variants provide a compact description of nominal energy input that is physically consistent with established links to melt-pool behavior and solidification conditions, without implying direct measurement of melt-pool stability or thermal gradients. These conditions are reflected downstream through pore population summaries (size, volume fraction, morphology) and prior-β grain morphology (aspect ratio and characteristic length scales), while stress–strain-derived descriptors serve as morphology-adjacent signatures of the combined imprint of microstructure, defects, and residual stress state.

This hierarchy is aligned with the target-dependent value of morphology-derived information observed in the modeling outcomes. Defect-sensitive properties, particularly yield strength and fatigue-relevant surface proxies, are expected to respond to pore volume statistics and sphericity distributions because pores act as stress concentrators and reduce the effective load-bearing area. Where the morphology module indicates usable embedding predictability, the predicted-embedding pathway suggests that parts of this defect-related signal are recoverable from process variables in the present dataset, consistent with the role of energy input and scan strategy in controlling lack-of-fusion and keyhole regimes. More modest or target-specific gains from morphology augmentation should not be interpreted as evidence that the prior-β grain descriptors are irrelevant; rather, the grain-summary block captures only one part of the latent microstructural state, whereas the stress–strain-derived block reflects the integrated response of prior-β morphology together with pores, phase constitution, texture, residual stress, and lattice-defect/dislocation substructure, so only partial correlations between the two are expected.

### 6.2. What the Multimodal Gains Mean for Nanomaterials-Enabled Functional Surfaces and AM Process Tuning

The multimodal outcomes carry two implications for functional surfaces and process tuning under the present feature and label regime [31,32,33,34]. First, the oracle-versus-deployable comparison provides an explicit bound on how much morphology information can improve property prediction when morphology is fully available versus when it must be inferred from deployable inputs. Oracle morphology embeddings represent the idealized upper bound in which pore and grain descriptors are available at inference time, whereas predicted morphology embeddings correspond to the deployable setting in which morphology is inferred from process settings. The gap between these conditions quantifies how much of the morphology signal is structurally learnable from process variables alone in the current dataset; a small gap is consistent with treating morphology as an implicit latent state recoverable from process descriptors for prospective screening, whereas a large gap indicates irreducible morphology information not encoded in process variables and therefore motivates targeted characterization of a minimal morphology panel that most improves decision-making.

Second, for surface-facing objectives as represented here by surface condition constraints and roughness-linked proxies, the co-design and risk-aware selection workflow supports joint tuning of bulk mechanical objectives and surface-relevant objectives under uncertainty. Even when direct surface or bio-proxy measurements are sparse, uncertainty-aware ranking reduces the likelihood of selecting candidates that appear optimal only due to model variance. The constraint-satisfaction analysis and Pareto visualization formalize tradeoffs central to functional implants and engineered interfaces, where high strength must coexist with acceptable roughness and surface condition constraints [35,36,37]. Practically, the workflow identifies process windows expected to deliver compliant bulk properties while maintaining surface-relevant bounds and highlights regimes where predicted uncertainty is high and additional experiments would be maximally informative.

### 6.3. Conclusions

This work presents a deployment-oriented surrogate modeling framework for L-PBF Ti-6Al-4V that explicitly reflects the constraints of small, set-level experimental datasets and partially observed morphology descriptors. By enforcing recipe-level grouping (GroupKFold by set_id) and fold-respecting preprocessing, the study provides a leakage-resistant basis for comparing process-only and multimodal predictors.

A key contribution is the principled separation of oracle multimodal inference (morphology available at decision time) from a deployable setting in which morphology must be inferred from process-accessible inputs via a two-stage embedding strategy. Across targets, the resulting models achieve strong performance for primary mechanical properties while underscoring that roughness and hardness remain difficult, likely reflecting label sparsity and missing or weakly captured surface-state determinants. Finally, the framework operationalizes uncertainty through fold-ensemble variability and integrates this signal into a constraint-aware co-design workflow, enabling conservative screening and recommendation of candidate recipes within the feasible process envelope. Collectively, the study advances a reproducible and practically actionable template for data-scarce AM optimization, bridging multimodal learning and decision-making under uncertainty. More broadly, the present framework is consistent with recent efforts in other safety-critical engineering domains to combine physically informed modeling, hybrid learning, and calibrated uncertainty estimation to improve deployment trustworthiness [37].

Although developed here for L-PBF Ti-6Al-4V, the framework is general to other process–structure–property systems with small, partially observed multimodal datasets. Extension to a new alloy or manufacturing route would require redefinition of the relevant process descriptors, morphology/microstructure proxies, and targets, followed by system-specific retraining and prospective validation of the surrogate and embedding models.

### 6.4. Data and Code Availability

The base dataset underpinning this study is publicly available and can be obtained from Zenodo (record 6587905): https://zenodo.org/records/6587905 (accessed on 31 March 2026) [38]. Derived datasets and research artifacts produced in the course of this work, including curated set-level master tables, engineered physics features (e.g., line energy density and energy-density variants), morphology and microstructure summary features (pore and prior-β grain descriptors), stress–strain-derived descriptors, finalized feature filters, GroupKFold split definitions, trained-model outputs (fold-level predictions), and aggregated performance summaries, have not been deposited in a public repository. These derived materials are available from the corresponding author upon reasonable request and subject to any applicable data-use, privacy, or third-party restrictions.

The code used to generate the results reported in this study is not publicly available at this time, owing to practical constraints related to project-specific dependencies, environment configuration, and associated research artifacts. However, the code can be made available by the corresponding author upon reasonable request, subject to any applicable institutional, licensing, and third-party restrictions.

## Figures and Tables

**Table 1:** Dataset and GroupKFold split summary.

No.	Metric	Value
1	Total rows (recipes)	42
2	Independent sets (set_id)	42
3	set_id range	(1, 42)
4	Labeled sets: Yield strength (MPa)	42/42
5	Labeled sets: Ultimate tensile strength (MPa)	42/42
6	Labeled sets: Elongation (%)	42/42
7	Labeled sets: Elastic modulus (GPa)	42/42
8	Labeled sets: Roughness (µm)	28/42
9	Labeled sets: Hardness (HV)	42/42
10	GroupKFold fold 1: train sets	33
11	GroupKFold fold 1: test sets	9
12	GroupKFold fold 2: train sets	33
13	GroupKFold fold 2: test sets	9
14	GroupKFold fold 3: train sets	34
15	GroupKFold fold 3: test sets	8
16	GroupKFold fold 4: train sets	34
17	GroupKFold fold 4: test sets	8
18	GroupKFold fold 5: train sets	34
19	GroupKFold fold 5: test sets	8

**Table 2:** Locked modeling, preprocessing, and decision-layer settings.

No.	Component	Setting	Specification
1	Global preprocessing	Unit of independence (grouping)	set_id
2	Global preprocessing	Split protocol	Grouped K-fold cross-validation (set-level; manual_group_kfold_like)
3	Global preprocessing	Missing-data handling	Median imputation for numeric features (fit on training data only)
4	Global preprocessing	Feature scaling	Standardization applied only where required, fit on training data only
5	Morphology module	Reproducibility control	Locked configuration
6	Co-design	Reproducibility control	Locked configuration

**Table 3:** Fold-aggregated predictive performance under 5-fold GroupKFold evaluation.

Target	Model	RMSE (Mean ± Std)	MAE (Mean ± Std)	R^2^ (Mean)
Yield strength (MPa)	XGBoost	11.069 ± 8.677	7.358 ± 3.820	0.895
Ultimate tensile strength (MPa)	XGBoost	13.883 ± 12.060	8.473 ± 3.937	0.873
Elongation (%)	XGBoost	0.677 ± 0.499	0.489 ± 0.287	0.861
Elastic modulus (GPa)	XGBoost	2.379 ± 0.796	1.681 ± 0.338	0.663
Surface roughness (µm)	XGBoost	2.313 ± 0.816	1.951 ± 0.581	0.121
Vickers hardness (HV)	XGBoost	16.537 ± 4.658	13.755 ± 3.825	0.114

**Table 4:** Uncertainty-aware co-design recommendations under constraints.

Rank	P (W)	V (mm/s)	LED (J/m)	Yield (MPa)	Yield LCB (MPa)	UTS (MPa)	Elong. (%)	Modulus (GPa)	Roughness (µm)	Roughness UCB (µm)	Hardness (HV)	Score
1	131.8	781.8	297.2	1011.3 ± 7.2	1005.8	1082.0 ± 5.7	9.28 ± 0.23	111.35 ± 1.82	18.02 ± 1.97	19.50	365.9 ± 3.8	0.902
2	238.1	1167.7	237.4	1018.4 ± 6.8	1013.3	1080.3 ± 9.2	9.33 ± 0.27	107.21 ± 1.82	18.52 ± 1.03	19.29	365.6 ± 4.0	0.876
3	289.2	999.0	190.4	1018.6 ± 11.7	1009.8	1079.8 ± 10.8	9.51 ± 0.26	107.77 ± 1.48	18.52 ± 1.51	19.66	376.5 ± 7.9	0.866
4	200.4	842.6	228.8	1010.6 ± 7.5	1005.0	1083.3 ± 5.6	9.22 ± 0.23	114.18 ± 0.43	17.09 ± 1.41	18.15	379.1 ± 8.6	0.856
5	207.4	850.0	194.8	1011.0 ± 8.0	1005.0	1090.7 ± 4.5	9.27 ± 0.40	113.08 ± 0.96	18.52 ± 1.12	19.36	393.5 ± 3.1	0.856
6	143.3	1530.9	215.6	1023.1 ± 12.7	1013.6	1080.0 ± 10.1	9.36 ± 0.21	106.32 ± 2.26	16.93 ± 1.48	18.05	389.8 ± 7.3	0.853
7	376.8	1294.3	261.5	1013.9 ± 4.5	1010.5	1081.4 ± 1.6	8.43 ± 0.28	111.22 ± 0.84	16.86 ± 1.14	17.72	369.6 ± 2.2	0.835
8	332.1	446.1	251.4	1005.9 ± 7.4	1000.3	1074.9 ± 3.3	9.12 ± 0.11	107.32 ± 1.96	19.10 ± 1.05	19.89	370.5 ± 3.4	0.782
9	340.1	868.1	445.4	1013.1 ± 12.1	1004.0	1081.7 ± 9.6	8.18 ± 0.12	109.34 ± 0.67	17.50 ± 1.40	18.55	371.5 ± 5.2	0.765
10	434.2	941.0	296.6	1006.1 ± 2.2	1004.4	1073.2 ± 4.5	8.99 ± 0.23	107.43 ± 1.78	17.16 ± 1.22	18.08	383.7 ± 5.8	0.764
11	196.3	900.1	327.8	1004.3 ± 11.7	995.6	1075.5 ± 5.9	9.24 ± 0.23	108.43 ± 1.61	16.46 ± 1.83	17.83	385.3 ± 4.6	0.742
12	327.5	1456.3	362.2	1006.2 ± 9.0	999.5	1078.5 ± 4.7	8.80 ± 0.49	107.47 ± 1.81	18.08 ± 1.58	19.27	381.2 ± 5.7	0.740
13	376.7	1330.1	412.5	1003.8 ± 10.8	995.8	1072.2 ± 6.1	9.18 ± 0.26	108.89 ± 1.01	17.42 ± 0.70	17.94	387.4 ± 5.6	0.702
14	256.0	679.0	322.6	1005.8 ± 12.4	996.5	1076.3 ± 8.3	8.92 ± 0.28	107.11 ± 1.86	18.12 ± 1.83	19.49	369.6 ± 6.6	0.682
15	369.0	1288.4	343.4	994.9 ± 8.0	988.9	1073.0 ± 3.6	9.23 ± 0.27	113.12 ± 1.44	19.48 ± 0.86	20.13	370.2 ± 5.1	0.640
16	493.2	863.6	274.1	1002.6 ± 8.3	996.4	1069.4 ± 4.9	8.77 ± 0.33	107.71 ± 1.84	17.60 ± 1.65	18.84	370.9 ± 4.6	0.620
17	213.9	1138.3	288.3	991.6 ± 5.0	987.9	1070.2 ± 2.2	8.95 ± 0.12	112.86 ± 1.61	18.46 ± 1.73	19.75	385.8 ± 7.1	0.597
18	481.5	1346.8	232.0	998.8 ± 4.1	995.7	1058.3 ± 3.6	9.29 ± 0.23	110.85 ± 0.98	17.70 ± 2.23	19.37	371.9 ± 1.3	0.573
19	449.6	496.5	194.3	996.3 ± 11.1	987.9	1074.8 ± 5.7	8.80 ± 0.54	106.83 ± 1.74	16.13 ± 1.58	17.31	365.7 ± 4.6	0.573
20	430.8	1469.2	391.6	994.7 ± 5.8	990.4	1070.2 ± 4.1	8.77 ± 0.23	113.82 ± 0.65	16.23 ± 1.11	17.06	381.9 ± 2.5	0.562
21	478.5	1378.9	464.6	985.6 ± 8.1	979.6	1070.4 ± 4.3	9.18 ± 0.25	109.08 ± 1.38	18.70 ± 1.61	19.91	369.7 ± 4.3	0.555
22	212.0	559.5	265.7	1002.4 ± 8.2	996.3	1059.1 ± 6.2	9.29 ± 0.20	107.19 ± 1.84	17.93 ± 1.05	18.71	378.3 ± 2.2	0.546
23	367.5	1042.8	372.1	984.2 ± 9.7	976.9	1069.0 ± 2.7	9.16 ± 0.18	113.30 ± 0.80	17.98 ± 1.28	18.95	365.1 ± 3.8	0.492
24	444.9	760.0	458.1	987.4 ± 7.2	982.1	1065.3 ± 6.2	9.17 ± 0.34	110.75 ± 0.76	18.07 ± 1.82	19.44	381.0 ± 5.7	0.489
25	445.7	1414.2	324.1	991.7 ± 8.3	985.5	1068.4 ± 3.7	8.13 ± 0.18	110.20 ± 0.49	18.64 ± 1.29	19.60	386.6 ± 13.6	0.486
26	268.9	1437.9	317.8	991.4 ± 7.2	986.0	1058.5 ± 4.8	9.34 ± 0.43	107.27 ± 1.63	17.63 ± 2.10	19.21	378.2 ± 7.4	0.448
27	365.2	997.4	234.2	1002.0 ± 8.4	995.6	1056.5 ± 6.6	8.72 ± 0.38	107.45 ± 1.84	18.37 ± 1.49	19.48	379.6 ± 6.8	0.436
28	342.0	1452.0	265.3	989.6 ± 5.1	985.8	1056.7 ± 4.9	9.32 ± 0.23	112.47 ± 0.74	18.45 ± 1.93	19.90	381.2 ± 5.3	0.435
29	294.1	918.6	461.1	984.9 ± 6.7	979.8	1067.1 ± 2.0	8.25 ± 0.12	107.73 ± 1.69	16.66 ± 0.96	17.38	364.8 ± 3.9	0.431
30	271.1	1088.6	223.5	996.6 ± 12.8	987.0	1067.4 ± 6.9	8.23 ± 0.18	113.47 ± 0.23	18.90 ± 1.56	20.07	369.1 ± 6.8	0.426
31	493.2	1420.2	447.5	987.3 ± 5.8	982.9	1065.8 ± 2.6	7.81 ± 0.12	110.14 ± 0.53	17.47 ± 1.78	18.81	370.8 ± 6.0	0.417
32	267.8	606.1	278.9	988.6 ± 3.1	986.2	1054.5 ± 4.0	9.06 ± 0.37	110.51 ± 0.85	17.05 ± 1.38	18.08	365.9 ± 1.8	0.410
33	254.7	1484.7	351.3	981.9 ± 10.1	974.3	1071.3 ± 3.1	8.15 ± 0.02	113.31 ± 0.43	17.75 ± 2.02	19.26	382.1 ± 9.7	0.397
34	201.9	825.4	242.9	992.0 ± 3.7	989.2	1052.0 ± 3.9	8.97 ± 0.12	107.15 ± 1.96	17.13 ± 1.63	18.35	383.7 ± 9.3	0.393
35	486.2	819.4	273.5	986.6 ± 12.1	977.5	1069.7 ± 4.8	8.13 ± 0.24	112.68 ± 1.08	16.21 ± 1.32	17.20	380.5 ± 6.7	0.381
36	319.3	1233.0	296.3	983.2 ± 3.3	980.8	1054.0 ± 2.8	9.32 ± 0.38	112.56 ± 0.47	16.33 ± 0.53	16.74	372.6 ± 3.0	0.365
37	223.3	1289.8	202.8	982.0 ± 8.9	975.3	1068.5 ± 4.1	7.95 ± 0.19	111.79 ± 1.00	15.49 ± 0.90	16.16	375.1 ± 7.5	0.352
38	321.3	625.7	255.1	993.4 ± 5.9	989.0	1047.5 ± 2.3	9.07 ± 0.36	107.20 ± 1.81	18.68 ± 1.56	19.85	372.6 ± 3.9	0.346
39	378.6	666.1	309.3	985.6 ± 2.2	984.0	1050.6 ± 4.5	9.20 ± 0.17	106.93 ± 1.86	17.10 ± 0.77	17.68	366.5 ± 4.1	0.343
40	212.1	809.2	404.8	982.4 ± 8.1	976.3	1055.6 ± 5.1	9.35 ± 0.28	107.32 ± 1.87	18.15 ± 1.33	19.15	379.9 ± 4.0	0.339
41	458.1	715.4	415.5	991.8 ± 6.2	987.2	1059.1 ± 4.8	8.09 ± 0.22	113.53 ± 0.42	18.87 ± 1.61	20.07	371.3 ± 3.4	0.331
42	236.1	713.3	333.4	981.6 ± 4.6	978.2	1051.6 ± 2.0	9.20 ± 0.39	107.25 ± 1.69	18.26 ± 1.36	19.29	376.6 ± 7.6	0.311
43	278.1	1472.9	338.9	988.8 ± 7.1	983.6	1051.2 ± 6.4	9.10 ± 0.30	107.14 ± 1.95	18.26 ± 1.38	19.29	374.5 ± 5.7	0.311
44	356.8	944.6	347.8	976.7 ± 16.8	964.1	1069.6 ± 10.9	9.05 ± 0.19	112.60 ± 0.51	18.80 ± 2.18	20.44	370.4 ± 3.2	0.311
45	200.3	437.8	252.4	992.0 ± 7.8	986.2	1047.0 ± 2.5	9.15 ± 0.26	113.69 ± 0.40	17.93 ± 1.19	18.83	384.2 ± 9.9	0.311
46	344.9	1082.9	403.6	977.2 ± 8.2	971.0	1067.7 ± 2.2	7.96 ± 0.34	107.44 ± 1.84	17.42 ± 1.41	18.48	386.6 ± 10.6	0.309
47	411.2	1573.7	393.9	974.9 ± 10.9	966.8	1065.1 ± 10.2	9.03 ± 0.36	109.44 ± 0.53	18.16 ± 1.71	19.44	388.3 ± 6.1	0.309
48	343.1	1564.8	254.9	997.4 ± 5.3	993.4	1048.6 ± 4.1	7.88 ± 0.28	109.39 ± 0.93	18.86 ± 1.51	19.99	381.8 ± 7.0	0.299
49	179.5	498.2	268.9	976.0 ± 12.4	966.7	1064.3 ± 7.8	9.17 ± 0.31	113.74 ± 0.81	17.80 ± 1.65	19.04	372.1 ± 2.2	0.289
50	155.2	1131.5	437.1	973.5 ± 5.2	969.6	1056.0 ± 5.6	9.26 ± 0.26	113.09 ± 0.60	17.32 ± 1.93	18.77	382.4 ± 8.2	0.259

## Data Availability

No new data were created or analyzed in this study. The data used in this study are publicly available on Zenodo (record 6587905).

## Appendix A. Dataset, Feature Engineering, and Modality Metadata

### Appendix A.1. Scope and Rationale

This appendix specifies the dataset schema, feature construction rules, modality definitions, and missing-data handling in a level of detail sufficient for strict computational reproducibility. The emphasis is placed on (i) unambiguous identifier logic at the set level, (ii) explicit feature semantics and units for both process-derived and morphology-derived descriptors, and (iii) consistent policies for filtering and imputation under partially observed multimodal measurements.

#### Dataset Schema and Identifiers

The analysis is conducted at the set level, indexed by a unique identifier denoted set_id, such that each set corresponds to a single experimental condition under which L-PBF process parameters, engineered physics descriptors, morphology or microstructure proxies, and property targets are coherently assigned. Raw inputs are heterogeneous and may be represented by multiple modality-specific files per set; consequently, a deterministic mapping from each raw file to set_id is enforced prior to aggregation. After parsing and mapping, all modalities are reduced to a single fixed-length set-level representation by modality-specific summarization operators and then joined into a unified master table keyed by set_id. This design ensures that each learning instance represents a complete experimental unit and prevents inadvertent replication of the same set across multiple rows.

### Appendix A.2. Target Definitions and Unit Conventions

Six targets are modeled as set-level scalar responses: yield strength (MPa), ultimate tensile strength (MPa), elongation to failure (%), elastic modulus (GPa), surface roughness (µm), and Vickers microhardness (HV). Targets are defined by explicit column identifiers within the processed master table, and the associated unit conventions are treated as fixed. Any transformation applied to targets, including clipping, winsorization, log transformation, or outlier removal, must be declared explicitly to maintain interpretability of performance metrics and comparability across studies; when no such transformations are applied, this absence is stated to preclude ambiguity. Targets with incomplete labeling are retained under a labeled-subset protocol, in which folds are evaluated using only those sets with valid target values, while preserving the set_id grouping constraint described in Appendix A.6.

### Appendix A.3. Tabular and Process-Derived Features with Engineered Physics Descriptors

Tabular predictors comprise the retained L-PBF process parameters and a set of engineered physics descriptors derived deterministically from those parameters. Process variables are retained subject to a coverage criterion, whereby a feature is preserved only if its non-null fraction meets a pre-specified threshold (reported below). Engineered descriptors include line-energy density and energy-density variants, which provide physically motivated compressions of the process space and facilitate cross-feature comparisons. For clarity and reproducibility, engineered features are defined by explicit formulae and units. A representative formulation for line-energy density is LED=P/v, where P denotes laser power and v denotes scan speed, with units consistent with the input parameterization. A representative volumetric energy density variant may be expressed as VED=P/(v h t), where h denotes hatch spacing, and t denotes layer thickness. Because such descriptors can be highly collinear with their constituent process parameters, collinearity is treated as an expected property of the feature space rather than an anomaly; downstream learning therefore emphasizes robust cross-validated performance under a fixed feature specification rather than relying on ad hoc feature pruning.

### Appendix A.4. Morphology and Microstructure Proxies: Pore Morphology, Prior-β Grain Morphology, and Stress–Strain Descriptors

Morphology and microstructure proxies are represented as modality-specific feature blocks extracted from raw modality files and summarized to the set level. Each modality is identified through a modality-aware inventory step that assigns files to one of the following categories: pore morphology, prior-β grain morphology, and stress–strain-derived descriptors. After modality identification and set_id mapping, each modality is summarized by a consistent family of distributional operators applied to per-object or per-curve measurements to produce a fixed-length descriptor vector per set. Specifically, for a raw measurement vector x within a set and modality, the summarization operator returns a standardized collection of statistics including sample count n, mean μ, standard deviation σ, extrema (min, max), and selected quantiles (for example, median and upper-tail quantiles), computed using a consistent quantile definition across all sets. Feature names encode the modality, the underlying physical quantity, and the aggregation operator, thereby enabling immediate traceability from any derived feature to its modality origin and statistical meaning.

For pore morphology, the raw measurements typically include pore volume, equivalent diameter, surface area, and sphericity, among others; the set-level representation is obtained by aggregating these distributions and, where appropriate, incorporating stabilized transforms (such as log-scale variants for heavy-tailed size distributions). For prior-β grain morphology, the raw measurements commonly include grain aspect ratio and equivalent diameter; these are summarized using the same aggregation family to preserve cross-modality consistency. For stress–strain-derived descriptors, curve-derived proxies are treated as a morphology-adjacent modality when they are used as input predictors; the resulting descriptors are computed deterministically from curve traces and summarized per set, yielding a stable set-level block that can be concatenated with process and morphology features under the locked specification. When a modality is absent for a subset of sets, the absence is treated explicitly as missingness in the corresponding feature block rather than as an implicit exclusion of those sets from the study.

### Appendix A.5. Missingness Characterization, Imputation Policy, and Feature Filtering

The multimodal structure implies non-uniform modality availability across sets, with certain sets lacking pore, grain, or stress–strain descriptors depending on upstream data availability. Missingness is quantified at both the feature level and the modality-block level to separate sporadic missingness from structurally absent modalities. Feature filtering is applied prior to model fitting using a coverage threshold defined as a minimum required non-null fraction across sets (for example, a criterion of at least 0.70 non-null coverage), ensuring that the retained feature set does not contain systematically unobserved predictors. For the remaining missing values within retained features, imputation is performed using a fold-respecting rule in which imputation parameters are estimated on the training portion of each fold and applied to the corresponding validation or test portion. Median imputation is adopted as the default policy because it is robust under heavy-tailed distributions and small-sample settings and does not impose parametric assumptions that are rarely defensible in sparse multimodal materials datasets. This fold-conditional approach prevents information leakage by disallowing test-fold statistics from influencing training-fold preprocessing.

### Appendix A.6. Final Locked Feature Sets and Reproducible Evaluation Protocol

All reported models are evaluated under locked feature specifications that define the exact set of retained predictors and the modality composition of each variant. Variants are defined by the concatenation of tabular/process features with one or more morphology blocks, including tab-only, tab plus pores, tab plus grains, tab plus pores and grains, and a combined multimodal variant containing all eligible morphology blocks. The locked specification includes both the prefiltering coverage criterion and the resulting postfilter feature list, thereby eliminating feature drift and ensuring that results can be reproduced exactly from the same processed master table. Model assessment follows a group-aware cross-validation protocol in which splits are constructed by GroupKFold with five folds, using set_id as the grouping variable, and where any hyperparameter selection, preprocessing parameter estimation, and imputation are conducted strictly within the training partition of each fold. This protocol enforces leakage control by construction, since no set_id appears simultaneously in training and test partitions within a fold, and it preserves the correct experimental unit of analysis throughout the pipeline.

## Appendix B. Modeling Protocol, Ablations, and Additional Diagnostics

This appendix specifies the modeling workflow in sufficient detail to enable independent reproduction while preserving concision in the main text. Emphasis is placed on leakage-safe evaluation for small-n, transparent model selection, and reporting practices that remain stable under partially missing modalities and targets.

### Appendix B.1. Cross-Validation Protocol and Leakage Controls

All predictive models are evaluated under a 5-fold GroupKFold protocol in which the grouping variable is the set-level identifier, set_id, such that no set contributes samples to both training and test partitions within a fold. The normalized split file contains five folds spanning all 42 sets, with fold sizes of train/test = 33/9 for folds 1–2 and 34/8 for folds 3–5. Leakage controls are enforced by performing every data-dependent operation strictly within each training fold and then applying the learned transformation to the corresponding test fold; this includes median imputation parameters, standardization parameters for scale-sensitive models, and any feature filtering rules based on non-null coverage. For the roughness target, only 28 of 42 sets are labeled; evaluation is therefore performed on the labeled subset within each fold’s held-out partition, and folds contribute to the reported metrics through the labeled test instances only (thereby preventing any imputed label leakage and avoiding artificial inflation of performance).

### Appendix B.2. Models and Hyperparameter Grids

Baseline comparisons include linear, kernel, neighborhood, and tree-based regressors, selected to represent widely used tabular learning families under CPU constraints. Hyperparameters are tuned via discrete grids using training-fold internal validation, and the selected best setting is recorded per fold and target. The Ridge regressor is tuned over alpha ∈ {0.1, 1.0, 10.0}. The SVR with RBF kernel is tuned over C ∈ {1, 10} and gamma ∈ {scale, 0.01}. KNN regression is tuned over n_neighbors ∈ {3, 7, 15}. Random Forest regression is tuned using a large forest (n_estimators = 2000) with max_depth ∈ {None, 5} and max_features ∈ {sqrt}. ExtraTrees regression is tuned using a large forest (n_estimators = 4000) with max_depth ∈ {None, 5} and max_features ∈ {sqrt}. Histogram-based Gradient Boosting is tuned with max_iter = 2000 and learning_rate ∈ {0.01, 0.05, 0.1}. XGBoost regression is tuned over learning_rate ∈ {0.02, 0.05, 0.1}, max_depth ∈ {3, 5}, and min_child_weight ∈ {1, 5}. LightGBM regression is tuned using n_estimators = 6000, learning_rate ∈ {0.02, 0.05}, max_depth ∈ {3, 5}, and min_child_samples ∈ {5, 20}. CatBoost regression is tuned using iterations = 6000, learning_rate ∈ {0.02, 0.05}, depth ∈ {4, 6, 8}, and l2_leaf_reg ∈ {10, 30}. Early stopping for boosting libraries is applied only when supported by the installed package API; otherwise, a fixed estimator budget is used, and model selection proceeds through the discrete grid described above to ensure version-robust reproducibility.

### Appendix B.3. Optimizer Ablation Details

The optimizer study evaluates training robustness for a fixed neural tabular regressor under multiple optimizers, with identical data splits and identical preprocessing across optimizers to isolate optimizer effects. The compared optimizers are AdamW, Muon (all-2D variant), and a hybrid strategy that applies Muon to selected submodules while retaining AdamW for the remaining parameters, thereby testing whether curvature-aware updates improve stability on small-n tabular regression. A log-spaced learning-rate sweep is conducted over a compact range spanning approximately 1 × 10^−4^ to 4 × 10^−2^, with weight decay included as a controlled regularizer; the selection rule is “fair” in the sense that each optimizer is permitted to operate at its fold-wise best learning rate, and fold-wise performance distributions are then summarized to quantify both central tendency and dispersion. Stability is reported using per-target boxplots of the validation metric across runs, and operating regions are summarized as target-by-learning-rate response maps to identify plateaus of stable training. The recommended final optimizer setting from the fairness study is AdamW with learning rate 1 × 10^−3^ and weight decay 1 × 10^−4^, which is used as the default for subsequent neural training runs when neural baselines are included.

### Appendix B.4. Metrics and Statistical Reporting

Predictive accuracy is quantified using RMSE, MAE, and coefficient of determination R^2^, computed on held-out test partitions within each fold. For a target vector y and predictions $\hat{y}$ over n held-out samples, RMSE is defined as $\sqrt{\frac{1}{n}\sum_{i=1}^{n}(y_{i}-{\hat{y}}_{i}{)}^{2}}$, MAE as $\frac{1}{n}\sum_{i=1}^{n}|y_{i}-{\hat{y}}_{i}|$, and $R^{2}$ as $1-\frac{\sum_{i}(y_{i}-{\hat{y}}_{i}{)}^{2}}{\sum_{i}(y_{i}-\bar{y}{)}^{2}}$. Reported headline values are the mean and standard deviation of fold-wise metrics computed across the five folds (or across the subset of folds contributing labeled test instances for partially labeled targets). Comparative statements between the final hybrid surrogate and the strongest baseline are based on fold-level deltas (final minus baseline) for RMSE and R^2^, and confidence intervals are computed via bootstrap resampling of folds with replacement using a percentile-based interval; the replicate count and random seed used for bootstrap resampling are recorded in the exported results bundle for exact reproducibility.

### Appendix B.5. Additional Plots and Checks

Supplementary diagnostics are used to assess failure modes beyond aggregate scalar metrics. First, parity plots are examined per target to identify systematic bias, slope compression, and heteroscedastic regions, with particular attention to targets exhibiting weak R^2^ (for example, hardness under purely tabular baselines). Second, residual-versus-predicted plots are generated to diagnose heteroscedasticity and leverage points that can dominate RMSE in small-n settings. Third, modality sensitivity checks are conducted by rerunning model selection under alternative feature-set definitions (tabular-only versus tabular plus individual morphology modalities) to quantify whether performance gains arise from genuine information content rather than feature-count inflation. Finally, robustness to feature filtering is evaluated qualitatively by confirming that the selected surrogate remains stable under reasonable non-null coverage thresholds (for example, 0.70 as the default), and any deviations are documented to prevent overinterpretation of single-threshold artifacts.

## Appendix C. Morphology Module and Co-Design Methodology

This appendix specifies the two-stage morphology strategy and the uncertainty-aware co-design pipeline in a deployment-feasible form. The objective is to formalize a procedure that remains statistically defensible under small sample size, multimodal heterogeneity, and partially missing modality coverage, while preserving reproducibility through explicit training, leakage control, and selection rules.

### Appendix C.1. Morphology Embedding Construction

Morphology information is represented through modality-specific low-dimensional embeddings derived from set-level summarized features for pores, prior-β grain morphology, and stress–strain-derived descriptors. Embeddings are constructed using principal component analysis (PCA) fit exclusively on training folds within the GroupKFold protocol, and then applied to the corresponding held-out fold; PCA is never fit on any data that includes the test fold, preventing leakage through global covariance structure. Dimensionality is controlled by a cumulative explained-variance threshold of 0.95 together with a hard cap of eight components, which constrains model flexibility and mitigates overfitting in the low-n regime. Under this procedure, the effective dimensionality collapses to one component per modality in the reported run (pores: 1, grains: 1, stress: 1), consistent with strong redundancy after aggregation and with the need for stable downstream regression. This embedding choice prioritizes (i) dimensionality discipline when the number of sets is small, (ii) fold-stable representations, and (iii) interpretability of modality contribution through a compact latent axis.

### Appendix C.2. Embedding Predictability Model

A supervised predictor is trained to infer morphology embeddings from tabular and process inputs, enabling morphology-aware inference at decision time when morphology measurements are unavailable. The embedding predictability model is trained and evaluated under the same GroupKFold partitions and preprocessing discipline as the primary surrogates: preprocessing statistics are estimated on training folds and applied to test folds, and any feature filtering or imputation is performed without access to the held-out fold. Predictability is quantified by out-of-fold R^2^ computed separately for each modality embedding and then summarized across folds; these metrics characterize the extent to which process conditions encode morphology variation and provide a deployment-relevant diagnostic of whether predicted morphology embeddings are informative or should be down-weighted in decision making.

### Appendix C.3. Oracle, Predicted, and Deployable Variants

Three model variants are defined to separate scientific upper bounds from deployment-feasible configurations. The oracle variant uses embeddings computed directly from measured morphology features and therefore assumes that full morphology characterization is available at inference time; this variant defines an optimistic ceiling but is not generally deployable. The predicted variant replaces measured embeddings with embeddings predicted from process parameters, maintaining a morphology-informed representation without requiring morphology measurements for new designs. The deployable variant restricts all inputs to those available at decision time and uses predicted embeddings only for targets whose surrogate specification requires morphology information; targets for which morphology does not improve performance or is not required remain purely tabular. Selection of the deployable variant per target follows an explicit exclusion rule that disallows oracle models and chooses the best non-oracle configuration under cross-validated performance, ensuring that reported co-design decisions are consistent with the deployment constraint rather than retrospective availability of measurements.

### Appendix C.4. Candidate Generation

Candidate recipes are generated by sampling within a physically plausible design space anchored to the observed process-parameter ranges, with optional controlled expansion if exploration beyond the experimental envelope is intended and explicitly justified. A fixed candidate budget is used for tractability, with 5000 candidates generated in the reported run. The design vector is defined by the filtered tabular and process feature set retained after coverage-based filtering, and engineered physics features are computed deterministically from sampled process parameters using the same feature definitions as in model training to preserve schema consistency. When a target’s deployable surrogate requires morphology information, candidate morphology embeddings are obtained by applying the embedding predictability model to candidate process features, thereby producing morphology-informed inputs without relying on unmeasured modalities. Feasibility safeguards are applied by enforcing bounds and admissibility checks on sampled variables, preventing candidates that violate physically meaningful limits or exceed the modeled operating space by construction.

### Appendix C.5. Uncertainty-Aware Constraint Satisfaction

Co-design is performed under predictive uncertainty using an ensemble derived from the fold-trained models. For each candidate, the ensemble mean μ is computed as the average prediction across the fold models, and the ensemble uncertainty σ is computed as the standard deviation across fold predictions, representing sensitivity to data resampling and model estimation in the small-n regime. Constraints are enforced through conservative risk-aware rules: for “must exceed” constraints, feasibility is assessed by μ − zσ ≥ τ, and for “must be below” constraints, feasibility is assessed by μ + zσ ≤ τ, where τ is the threshold and z sets the conservativeness. Because stringent conservativeness can eliminate feasible designs in partially observed and noisy regimes, an adaptive relaxation strategy is employed in which z and, where applicable, quantile requirements are iteratively reduced until a non-empty feasible set appears, while preserving a monotone relaxation schedule and logging each iteration. This procedure yields a transparent trade-off between reliability and feasibility, rather than an opaque single-shot thresholding that may fail silently.

### Appendix C.6. Recommendation Selection and Table 4 Construction

Recommendations are selected from the feasible set using multi-objective ranking appropriate to the design task. When competing objectives are present, Pareto filtering is applied to identify non-dominated candidates, followed by secondary ranking criteria that emphasize risk-adjusted performance under the same uncertainty-aware constraint rules. When objectives can be scalarized, a composite score is used with explicit weights and with penalties for uncertainty or constraint proximity if risk aversion is required. Table 4 reports the final recommendation set with process recipe variables defining each candidate, predicted means for the relevant targets, corresponding uncertainty estimates, explicit constraint pass/fail indicators under the adopted risk rule, and a rank or identifier supporting traceability. Physical validation requires fabrication of the recommended recipes within the specified bounds, measurement of the same mechanical and surface endpoints under consistent protocols, and quantitative comparison of measured outcomes against predictive means and conservative bounds to evaluate both accuracy and uncertainty calibration in a deployment-aligned setting.

## References

[1] Cao S., Zou Y., Lim C.V.S., Wu X. (2021). Review of laser powder bed fusion (LPBF) fabricated Ti-6Al-4V: Process, post-process treatment, microstructure, and property. Light Adv. Manuf..

[2] Ruckh E., Lambert-Garcia R., Hocine S., Getley A.C., Iantaffi C., Bhatt A., Fitzpatrick M., Marussi S., Farndell A., Schubert T. (2025). Process mapping of Ti-6Al-4V laser powder bed fusion using in situ high-speed synchrotron x-ray imaging. Addit. Manuf..

[3] Hassanin H., El-Sayed M.A., Ahmadein M., Alsaleh N.A., Ataya S., Ahmed M.M.Z., Essa K. (2023). Optimising surface roughness and density in titanium fabrication via laser powder bed fusion. Micromachines.

[4] Wang J., Zhu R., Liu Y., Zhang L. (2023). Online detection of balling phenomena for laser powder bed fusion using molten pool morphology. Adv. Powder Mater..

[5] Xu X., Xie Z., Wu M., Ma C. (2025). Effects of laser process parameters on melt pool thermodynamics, surface morphology and residual stress of laser powder bed-fused TiAl-based composites. Metals.

[6] Chowdhury S., Yadaiah N., Prakash C., Ramakrishna S., Dixit S., Gupta L.R., Buddhi D. (2022). Laser powder bed fusion: A state-of-the-art review of the technology, materials, properties & defects, and numerical modelling. J. Mater. Res. Technol..

[7] Dilip J.J.S., Zhang S., Teng C., Zeng K., Robinson C., Pal D., Stucker B. (2017). Influence of processing parameters on the evolution of melt pool, porosity, and microstructures in Ti-6Al-4V alloy parts fabricated by selective laser melting. Prog. Addit. Manuf..

[8] Qian C., Zhang K., Zhu J., Liu Y., Liu Y., Liu J., Liu J., Yang Y., Wang H. (2024). Effect of processing parameters on the defects distribution of Ti–6Al–4V alloy by selective laser melting. AIP Adv..

[9] Correa-Gómez E., Castro-Espinosa H., Caballero-Ruiz A., García-López E., Garcia Garcia G. (2024). Effect of process parameters on the roughness and tensile behavior of parts manufactured by the metals LPBF process. Eng. Rep..

[10] Hertlein N., Deshpande S., Venugopal V., Kumar M., Anand S. (2020). Prediction of selective laser melting part quality using hybrid Bayesian network. Addit. Manuf..

[11] Tapia G., Khairallah S.A., Matthews M.J., King W.E., Elwany A. (2018). Gaussian process-based surrogate modeling framework for process planning in laser powder-bed fusion additive manufacturing of 316L stainless steel. Int. J. Adv. Manuf. Technol..

[12] Tapia G., Elwany A.H. Prediction of porosity in SLM parts using a MARS statistical model and Bayesian inference. Proceedings of the Solid Freeform Fabrication Symposium (SFF).

[13] Tapia G., Elwany A.H., Sang H. (2016). Prediction of porosity in metal-based additive manufacturing using spatial Gaussian process models. Addit. Manuf..

[14] Montalbano T., Nimer S., Daffron M., Croom B., Ghosh S., Storck S. (2025). Machine learning enabled discovery of new L-PBF processing domains for Ti-6Al-4V. Addit. Manuf..

[15] Venkatachalam E., Sundararajan D. (2025). A review on the impact of volumetric energy density on morphological and mechanical behavior in laser powder bed fusion steel alloys. Weld. World.

[16] de Leon Nope G.V., Perez-Andrade L.I., Corona-Castuera J., Espinosa-Arbelaez D.G., Muñoz-Saldaña J., Alvarado-Orozco J.M. (2021). Study of volumetric energy density limitations on the IN718 mesostructure and microstructure in laser powder bed fusion process. J. Manuf. Process..

[17] Wu R., Wang H., Chen H.-T., Carneiro G. (2024). Deep multimodal learning with missing modality: A survey. arXiv.

[18] Qing J., Couckuyt I., Dhaene T. (2022). A robust multi-objective Bayesian optimization framework considering input uncertainty. J. Glob. Optim..

[19] Rheude T., Eils R., Wild B. Cohort-Based Active Modality Acquisition. Under Review as a Conference Paper at ICLR 2026 (submitted 19 September 2025; Modified 11 February 2026). https://openreview.net/pdf?id=QK8Mbgvtat.

[20] Valancius M., Lennon M., Oliva J.B. Acquisition Conditioned Oracle for Nongreedy Active Feature Acquisition. Proceedings of the 41st International Conference on Machine Learning (ICML 2024).

[21] Senthilnathan A., Nath P., Mahadevan S., Witherell P. (2025). Surrogate modeling of microstructure prediction in additive manufacturing. Comput. Mater. Sci..

[22] Noguchi S., Inoue J. (2021). Stochastic characterization and reconstruction of material microstructures for establishment of process-structure-property linkage using the deep generative model. Phys. Rev. E.

[23] Wang Y., Cui Z., Li Y. Distribution-consistent modal recovering for incomplete multimodal learning. Proceedings of the IEEE/CVF International Conference on Computer Vision (ICCV).

[24] Zeng Z., Peng Z., Yang X., Shen W. Missing as masking: Arbitrary cross-modal feature reconstruction for incomplete multimodal brain tumor segmentation. Proceedings of the MICCAI 2024.

[25] Slotwinski J., Cooke A., Moylan S. (2012). Mechanical Properties Testing for Metal Parts Made via Additive Manufacturing: A Review of the State of the Art of Mechanical Property Testing.

[26] Cepeda-Jiménez C.M., Potenza F., Magalini E., Luchin V., Molinari A., Pérez-Prado M.T. (2019). Effect of energy density on the microstructure and texture evolution of Ti-6Al-4V processed by laser powder bed fusion. Mater. Charact..

[27] Feng S., Chen Z., Bircher B., Ji Z., Nyborg L., Bigot S. (2022). Predicting laser powder bed fusion defects through in-process monitoring data and machine learning. Mater. Des..

[28] DebRoy T., Wei H.L., Zuback J.S., Mukherjee T., Elmer J.W., Milewski J.O., Beese A.M., Wilson-Heid A., De A., Zhang W. (2018). Additive manufacturing of metallic components—Process, structure and properties. Prog. Mater. Sci..

[29] King W.E., Anderson A.T., Ferencz R.M., Hodge N.E., Kamath C., Khairallah S.A., Rubenchik A.M. (2015). Laser powder bed fusion additive manufacturing of metals; physics, computational, and materials challenges. Appl. Phys. Rev..

[30] Le Roux S., Salem M., Shabbar M., Vicharapu B., Muthu N., Moussaoui K., Hor A., Velay V. (2025). Effect of process parameters on surface integrity in laser powder bed fusion of Ti-6Al-4V alloy. Sci. Rep..

[31] Gaikwad A., Giera B., Guss G.M., Forien J.-B., Matthews M.J., Rao P. (2020). Heterogeneous sensing and scientific machine learning for quality assurance in laser powder bed fusion—A single-track study. Addit. Manuf..

[32] Bhatt A., Huang Y., Leung C.L.A., Soundarapandiyan G., Marussi S., Shah S.M., Atwood R.C., Fitzpatrick M.E., Tiwari M.K., Lee P.D. (2024). In situ characterisation of surface roughness and its amplification during multilayer single-track laser powder bed fusion additive manufacturing. Addit. Manuf..

[33] Dareh Baghi A., Nafisi S., Hashemi R., Ebendorff-Heidepriem H., Ghomashchi R. (2023). A new approach to empirical optimization of laser powder bed fusion process for Ti6Al4V parts. J. Mater. Eng. Perform..

[34] Fullington D., Yangue E., Bappy M.M., Liu C., Tian W. (2024). Leveraging small-scale datasets for additive manufacturing process modeling and part certification: Current practice and remaining gaps. J. Manuf. Syst..

[35] Wennerberg A., Albrektsson T., Chrcanovic B. (2018). Long-term clinical outcome of implants with different surface modifications. Eur. J. Oral Implantol..

[36] Pegues J., Roach M., Williamson R.S., Shamsaei N. (2018). Surface roughness effects on the fatigue strength of additively manufactured Ti-6Al-4V. Int. J. Fatigue.

[37] Hossain R.B., Al Hasan M.M., Khan M.I., Ahmed M., Lin Y., Pan X. (2026). A physics-informed hybrid ensemble for robust and high-fidelity temperature forecasting in PMSMs. World Electr. Veh. J..

[38] Luo Q., Lu Y., Simpson T.W., Beese A.M. (2022). Effect of processing parameters on pore structures, grain features, and mechanical properties in Ti-6Al-4V by laser powder bed fusion. Addit. Manuf..

